# Little Cigar and Cigarillo Graphic Health Warnings and Quitting Behaviors: A Randomized Clinical Trial

**DOI:** 10.1001/jamanetworkopen.2025.26799

**Published:** 2025-08-15

**Authors:** Adam O. Goldstein, Kristen L. Jarman, Leah M. Ranney, Jennifer Cornacchione Ross, Sarah D. Kowitt, Chineme Enyioha, Sonia A. Clark, Paschal Sheeran, James F. Thrasher, Desmond Jenson, Nadja A. Vielot

**Affiliations:** 1 Department of Family Medicine, University of North Carolina at Chapel Hill; 2 Lineberger Comprehensive Cancer Center, University of North Carolina at Chapel Hill; 3 Department of Health Law, Policy, and Management, School of Public Health, Boston University, Boston, Massachusetts; 4 Department of Psychology and Neuroscience, University of North Carolina at Chapel Hill; 5 Department of Health Promotion, Education, and Behavior, Arnold School of Public Health, University of South Carolina, Columbia; 6 Public Health Law Center, Mitchell Hamline School of Law, St Paul, Minnesota

## Abstract

**Importance:**

Although little cigars and cigarillos (LCCs) are common combustible tobacco products, the effects of LCC warning labels on behavioral outcomes remain untested.

**Objective:**

To evaluate the effects of LCC graphic health warnings (GHWs) on promoting LCC quitting intentions and behaviors.

**Design, Setting, and Participants:**

This randomized clinical trial included 1029 adult LCC users from a Qualtrics online panel who were randomized to (1) LCC GHWs (n = 339), (2) existing US Food and Drug Administration (FDA) text-only warnings (n = 346), or (3) no stimulus control (n = 344). Participants accessed messages via email from May 1 to August 31, 2023, and completed surveys each day, with repeated exposure to LCC packages based on study condition.

**Interventions:**

Participants were exposed to 6 new LCC GHWs or FDA text-only cigar warnings 18 times over 3 weeks. The control group received no visual stimuli.

**Main Outcomes and Measures:**

The primary outcome was postintervention quit intentions, measured by a score ranging from 1 (low quit intention) to 4 (high quit intention). Secondary outcomes were the proportion having forgone or butted out (ie, extinguished an LCC that they had already lit) LCCs in the past week and the proportion having attempted to quit or reduced LCC use in the past week. Data were analyzed on an intent-to-treat basis using mixed-model analysis of variance and were adjusted for baseline behaviors.

**Results:**

Among the 1029 participants, the mean (SD) age was 43.9 (11.3) years, 578 (56.2%) were men, and 767 (74.5%) were users of both types of LCCs. Retention to trial end was high across all arms (LCC GHWs, 307 of 339 [90.6%]; FDA text-only warnings, 316 of 346 [91.3%]; and control group, 308 of 344 [89.5%]). Participants in the LCC GHW group reported significantly higher postintervention (and change from preintervention to postintervention) quit intentions (mean score, 2.9 [95% CI, 2.8-3.0]) compared with FDA text-only participants (mean, 2.5 [95% CI, 2.4-2.6]) and control (mean, 2.6 [95% CI, 2.4-2.7]) (*P* < .001). The mean proportion of participants reporting quit attempts after the intervention was significantly higher for participants in the LCC GHW group compared with participants in the text-only group (0.4 [95% CI, 0.4-0.5] vs 0.3 [95% CI, 0.2-0.3]). Participants in the LCC GHW group were more likely than those in the text-only group to forgo or butt out LCCs (adjusted estimate [SE]: forgoing little cigars, 0.12 [0.03]; forgoing cigarillos, 0.13 [0.03]; butting out little cigars, 0.07 [0.04]; butting out cigarillos, 0.07 [0.03]). No significant difference occurred in the change (from pre to post) in quit attempts between the LCC GHW and control groups.

**Conclusions and Relevance:**

This randomized clinical trial found that LCC GHWs were effective in increasing quit intentions and reducing LCC use behaviors. Current FDA warnings appear insufficient. Findings support enhanced GHWs on LCC packages to address significant public health gaps, aligned with global tobacco control strategies.

**Trial Registration:**

ClinicalTrials.gov Identifier: NCT05849051

## Key Points

**Question:**

Do graphic health warnings (GHWs) on little cigars and cigarillos (LCCs) improve intention to quit smoking and quitting behaviors?

**Findings:**

This 3-week randomized clinical trial of 1029 adults showed that those who smoked LCCs and were exposed to LCC GHWs reported significantly higher quitting behaviors compared with those exposed to existing US Food and Drug Administration text-only LCC warnings or a control condition.

**Meaning:**

This study provides the first evidence that LCC GHWs effectively increase quitting behaviors, offering support for policies mandating LCC GHWs to address public health concerns of LCC use in the US and internationally.

## Teaser

This randomized clinical trial evaluates the effects of graphic health warnings on little cigars and cigarillos on promoting quitting intentions and behaviors.

## Introduction

Cigar use is associated with significant health risks, including increased rates of cancer, respiratory diseases, and cardiovascular issues.^1,2^ Cigars, defined as any roll of tobacco wrapped in leaf tobacco rather than paper, have 3 major types: large or premium cigars that are hand rolled, little cigars that are cigarette sized with filters, and cigarillos that are smaller than premium cigars but larger than little cigars.^3^ Little cigars and cigarillos (LCCs) are the most frequently used cigar products, especially among young adults and people who are Black or African American, due to their affordability, flavors, and perceived reduced harm compared with cigarettes.^4,5^ Despite their health effects and the fact that cigars are not safe alternatives to cigarettes,^1^ LCCs are often overlooked in public health messaging for multiple reasons, including lowered risk perceptions, cultural and social norms of cigars being associated with wealth or luxury, foci on cigarettes or vaping, marketing loopholes, and regulatory gaps.^6,7,8^

Graphic health warnings (GHWs) on cigarettes, which visually depict the health consequences of smoking, have proven effective in reducing smoking intentions, reducing product appeal, and promoting quit attempts for cigarettes.^9,10,11^ Although the World Health Organization (WHO) Framework Convention on Tobacco Control has recommended GHWs, with more than 120 countries adopting them,^12^ almost all of the WHO GHWs relate to cigarettes; to our knowledge, longitudinal behavioral outcomes for GHWs for cigars are lacking.^12^

Preliminary evidence supporting GHWs for LCCs shows that compared with text-only warnings, GHWs elicit a negative affect and thinking about the risks of smoking among cigarillo smokers.^13^ However, the current US Food and Drug Administration (FDA) text-only warnings on cigars, including LCCs, appear insufficient in influencing short-term user perceptions and behavior.^14,15,16^ Progress has also been especially hindered by legal challenges, such as court rulings that struck down the FDA’s proposed mandatory cigar warnings, citing insufficient evidence to demonstrate the specific effect of such warnings on cigar use behaviors in the US.^17^ As a result, the current system for reducing LCCs via warnings in the US relies on voluntary text-only warnings, leaving a significant regulatory gap in efforts to effectively communicate the health risks of cigar smoking to consumers.^18^

The current legal precedent underscores a need for research to generate robust, context-specific evidence on the effectiveness of LCC GHWs. Researchers cannot assume that GHWs proven effective for cigarettes will work just as well for cigars due to several key differences in the products, users, and perceptions. For instance, cigarettes are typically smoked more frequently than cigars.^19^ Cigar users may perceive cigars are safe, even if not inhaled, making cigar users less responsive to GHWs designed for cigarette users.^1,14^ Finally, cigars are often associated with luxury and relaxation, with cultural images that might mitigate any discomfort induced by GHWs designed for cigarettes that are generally viewed as addictive and harmful.^20,21^

The present study fills this research gap by evaluating the longitudinal effect of LCC GHWs on LCC use through a health communication randomized clinical trial (RCT). The groundwork for the LCC GHWs builds on robust evidence from cigarette warning studies and the development of LCC warnings.^22,23,24,25^ The RCT examines whether LCC GHWs can effectively reduce intentions to use LCCs, increase quit attempts, and reduce use of these products.

## Methods

We preregistered our study at ClinicalTrials.gov (NCT05849051). The University of North Carolina institutional review board approved all study procedures, and all participants provided informed consent online prior to participation. This report followed the Consolidated Standards of Reporting Trials (CONSORT) reporting guideline for randomized clinical trials.^26^ The trial protocol is available in Supplement 1.

### Trial Design

We conducted a parallel, 3-condition RCT with the following conditions: (1) a hypothetical LCC label with GHWs developed by the study team at 30% size (LCC GHWs); (2) a hypothetical LCC label with existing, FDA text-only LCC warnings at 30% size (existing FDA text-only warnings); and (3) a control condition in which participants did not see warnings or LCC packages but were asked to complete daily surveys on previous-day LCC behaviors.

### Inclusion Criteria

We enrolled a national sample of US adults aged 21 years or older who reported currently using LCCs (ie, some days or every day). Eligibility included living in the US, having an email address and access to the internet at work or home, and agreeing to provide honest answers. We recruited participants from the online survey panel Qualtrics to participate in the trial from May 1 to August 31, 2023.

### Sample Size

The sample size was based on a power calculation using a previous quit intentions effect size from an RCT comparing graphic cigarette warnings with text-only warnings.^10^ Based on the effect size observed in that study, with a sample size of 750, we would have 80% power at α = .05 to detect a small change in primary outcome differences between our groups (Cohen *f* = 0.12).

### Research Procedures

**Figure 1.**
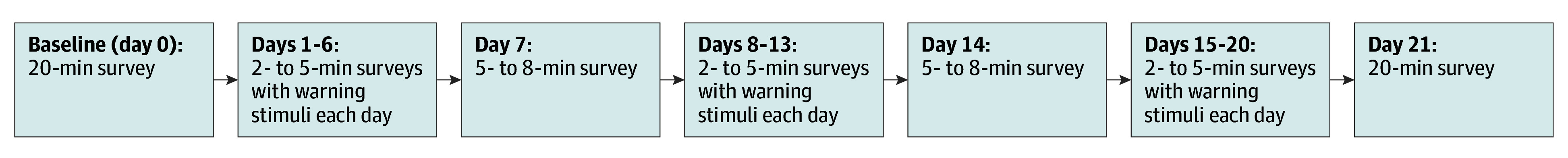
Study Participation Timeline

We contacted eligible participants through email to invite them to enroll in the survey by taking a baseline questionnaire. At the end of the baseline questionnaire, we randomized participants to the 3 conditions using a simple random assignment in the survey software. Subsequently, participants in the LCC GHW and FDA conditions completed a short survey, delivered through email each day for 6 consecutive days according to their assigned condition, on their LCC use and exposure to the stimuli after viewing a mock LCC package. No time limit existed for looking at the mock LCC package. Those in the control condition completed a short survey on their LCC use for 6 consecutive days. On the seventh day, participants completed a slightly longer survey assessing additional outcomes. This procedure was repeated 3 times, and the study ended on day 21 with a posttest survey (study timeline in Figure 1). Multiple strategies were implemented to encourage participants to complete the assigned surveys, including reaching out to participants via email with invitations and reminders and compensation up to $50 based on completion of questionnaires.

### Intervention

The groundwork for the LCC GHWs was initially established through studies identifying that health effects were the most effective themes for LCC GHWs.^15,22,24^ New LCC GHWs were created by empirically identifying optimal text statements and images to go with the health themes, demonstrating that depicting 2 health effects had a greater impact than 1 health effect, and combinations of external and internal pictorial representations of diseases outperformed either alone in terms of perceived message effectiveness and other related outcomes.^23,24^ A final short-term experiment showed that the LCC GHWs performed better than the LCC text warnings alone, and a label size of 30% of the warning on the pack performed as well as a label size of 50%, leading to the adoption of the 30% size.^25^

### Comparators

Six LCC GHWs were developed and compared with the 6 FDA text-only warnings. Under the LCC GHW and existing FDA text-only conditions, participants were shown mock LCC packages based on their assigned condition (trial protocol in Supplement 1). The 6 warnings were randomly assigned to 1 day of each week of the protocol, so throughout the study, participants in the LCC GHW and FDA text-only conditions could have seen each warning up to 3 times. Warning labels were shown on purple LCC packages that were branded with the fictitious name “Brentfield,” which has successfully been used in prior research to minimize the influence of brand loyalty and preexisting brand perceptions.^5^ Participants in the control condition did not receive any LCC package stimuli.

### Outcomes

The screening and baseline surveys assessed demographic characteristics as well as LCC product use, tobacco product use, nicotine dependence (assessed by the nicotine dependence score; range, 1-5, where 0-1 indicates low to no dependence, 2-3 indicates moderate dependence, and 4-5 indicates high dependence),^27^ tobacco beliefs, and previous warning exposure. Self-reported race and ethnicity included American Indian or Alaska Native, Asian, Black or African American, Latino or Hispanic, White, more than 1 race, unknown, or other race, ethnicity, or origin (including people who reported that their race is Middle Eastern or Northern African, Native Hawaiian or Other Pacific Islander, or other race, ethnicity, or origin). Race and ethnicity variables were modeled after Population Assessment of Tobacco and Health Study.^28^ We measured race and ethnicity in the study because LCC use rates are significantly higher among certain racial and ethnic groups, such as non-Hispanic Black adults, which may affect both exposure to and the impact of health warnings.

The primary outcome, LCC quit intentions, was measured at baseline and after the survey with 3 items: “How interested are you in quitting smoking cigars in the next 6 months?,” “How much do you plan to stop smoking cigars in the next 6 months?,” and “How likely are you to stop smoking cigars in the next 6 months?,” each on a scale of 4 (indicating very) to 1 (indicating not at all), varied to match the prompt.^29,30,31^ Responses to the 3 quit intention questions were averaged to create a score ranging from 1 (low quit intention) to 4 (high quit intention) for each participant.

The secondary outcomes were quitting behaviors, including quit attempts (“In the last 7 days, have you attempted to stop smoking cigars?”); butting out (ie, extinguishing a cigarillo that they had already lit) cigarillos (“In the last 7 days, how many times did you butt out a cigarillo before you finished because you wanted to smoke less?”); and forgoing cigarillos (“In the last 7 days, how many times did you stop yourself from having a cigarillo because you wanted to smoke less?”). Identical questions were asked for little cigars.

### Statistical Analysis

Data were analyzed on an intent-to-treat basis. We used mean (SD) values to describe continuous variables and percentages to describe categorical variables pertaining to the sample, including sociodemographic characteristics, LCC behaviors, and study retention. We inspected our data for patterns of missingness in postintervention (day 21) reports of quit intentions and quit attempts. We determined that missingness was arbitrary and did not differ by treatment assignment, and we used a fully conditional specification to impute missing outcome data based on an arbitrary missingness pattern and logistic regression of a combination of continuous, ordinal, and nominal variables. We generated 100 imputed datasets and estimated pooled results for the intervention effects on quit attempts and quit intentions. Pooled linear regression estimated the mean values for quit intentions and the proportion of participants who reported quit attempts, as well as the preintervention to postintervention changes in these outcomes by condition. A Bonferroni correction was applied to account for an inflated type I error rate associated with comparing 2 intervention groups with a common control group, with statistical significance set at a 2-sided α = .025.

We estimated the effect of the condition on butting out and forgoing LCCs on study days 7, 14, and 21 using mixed model analysis of variance with repeated measures to estimate the relative change in frequency of reported butting out and forgoing LCCs between treatment groups over time. Models were then adjusted for baseline behavioral level and interaction between condition assignment and study week in sensitivity analyses. All analyses were conducted in SAS, version 9.4 (SAS Institute Inc).

## Results

**Figure 2.**
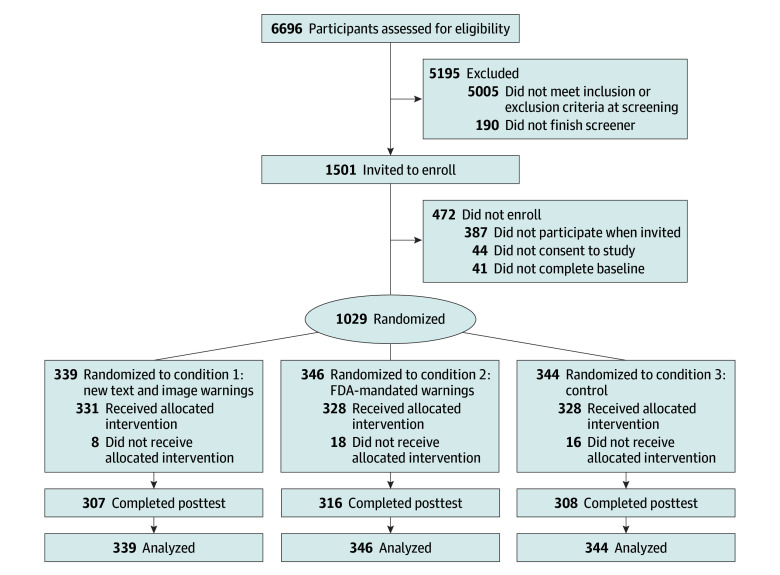
Flow Diagram for Recruitment and Enrollment Based on Consolidated Standards of Reporting Trials Reporting Guideline^26^ FDA indicates US Food and Drug Administration.

A total of 6696 individuals were assessed for study eligibility through Qualtrics, of whom 1501 were invited to enroll in the study. The primary reason that people were excluded from the study was because they did not currently use either little cigars or cigarillos (n = 4659). A total of 68.5% of invitees (n = 1029) provided informed consent to participate and were randomized into the 3 study conditions: LCC GHWs (n = 339), FDA text-only LCC warnings (n = 346), and no warning control (n = 344). At baseline, 889 cigarillo users reported smoking a mean of 1.8 cigarillos the previous day (range, 0-24 cigarillos), and 746 little cigar users reported smoking a mean of 1.3 little cigars the previous day (range, 0-21 little cigars). Retention to the end of the trial was high and similar across all arms (LCC GHWs, 307 of 339 [90.6%]; FDA text-only warnings, 316 of 346 [91.3%]; and control group, 308 of 344 [89.5%]). A flow diagram for recruitment, enrollment, and retention is shown in Figure 2.^26^

The total study sample was 1029 participants (mean [SD] age, 43.9 [11.3] years; 578 men [56.2%] and 449 women [43.6%]). The sample included 10 American Indian or Alaska Native individuals (1.0%), 57 Asian individuals (5.5%), 217 Black or African American individuals (21.1%), 170 Latino or Hispanic individuals, 645 White individuals (62.7%), 65 individuals of more than 1 race (6.3%), and 27 individuals of other race, ethnicity, or origin (2.6%). A total of 798 of 1028 individuals (77.6%) had completed at least some college education, 895 of 1025 (87.3%) lived above the federal poverty line, and 934 of 1028 (90.9%) identified as straight or heterosexual (Table 1). Most participants (n = 767 [74.5%]) were current users of both cigarillos and little cigars, whereas 220 (21.4%) reported using only cigarillos and 42 (4.1%) reported using only little cigars. The mean (SD) nicotine dependence score was 2.6 (1.7), indicating moderate dependence. Participants in all study arms reported seeing an existing warning label on an LCC package most of the time in the month prior to the study. Participants completed surveys on a mean (SD) of 14.8 (4.7) of 21 study days.

**Table 1.** Baseline Characteristics of Study Participants by Randomization Condition

Characteristic	LCC GHWs (n = 339)	FDA text-only LCC warnings (n = 346)	No warning control (n = 344)	Total sample (N = 1029)
Age, mean (SD), y	43.0 (10.4)	44.5 (11.7)	44.1 (11.6)	43.9 (11.3)
Gender, No. (%)				
Male	175 (51.6)	208 (60.1)	195 (56.7)	578 (56.2)
Female	163 (48.1)	138 (39.9)	148 (43.0)	449 (43.6)
Other or nonbinary	1 (0.3)	0	1 (0.3)	2 (0.2)
Race, No. (%)				
Asian	22 (6.5)	15 (4.3)	20 (5.8)	57 (5.5)
American Indian or Alaska Native	5 (1.5)	4 (1.2)	1 (0.3)	10 (1.0)
Black or African American	70 (20.7)	70 (20.2)	77 (22.4)	217 (21.1)
White	203 (59.9)	228 (65.9)	214 (62.2)	645 (62.7)
>1 Race	24 (7.1)	20 (5.8)	21 (6.1)	65 (6.3)
Other race, ethnicity, or origin^a^	14 (4.1)	5 (1.5)	8 (2.3)	27 (2.6)
Unknown	1 (0.3)	3 (0.9)	2 (0.6)	6 (0.6)
Ethnicity, No. (%)				
Latino or Hispanic	68 (20.1)	50 (14.4)	52 (15.1)	170 (16.5)
Non-Latino or non-Hispanic	270 (79.6)	294 (85.0)	291 (84.6)	855 (83.1)
Unknown	1 (0.3)	2 (0.6)	1 (0.3)	4 (0.4)
Education, No. (%)^b^				
<High school	7 (2.1)	5 (1.4)	6 (1.7)	18 (1.8)
Completed grade 12: GED certification or high school diploma	68 (20.1)	79 (22.9)	65 (18.9)	212 (20.6)
Some college	77 (22.7)	81 (23.5)	84 (24.4)	242 (23.5)
Associate’s degree	47 (13.9)	47 (13.6)	50 (14.5)	144 (14.0)
Bachelor’s degree	89 (26.3)	94 (27.3)	107 (31.1)	290 (28.2)
Graduate or professional degree	51 (15.0)	39 (11.3)	32 (9.3)	122 (11.9)
Poverty status, No. (%)^c^				
Below poverty line	39 (11.5)	48 (13.9)	43 (12.6)	130 (12.7)
Above poverty line	299 (88.5)	297 (86.1)	299 (87.4)	895 (87.3)
Sexual orientation, No. (%)^d^				
Straight or heterosexual	303 (89.4)	312 (90.4)	319 (92.7)	934 (90.9)
Gay, lesbian, bisexual, or other	36 (10.6)	33 (9.6)	15 (7.3)	94 (9.1)
Cigarillo smoking status, No. (%)				
Never user	5 (1.5)	7 (2.0)	5 (1.5)	17 (1.7)
Ever user	334 (98.5)	339 (98.0)	339 (98.5)	1012 (98.3)
Current (some day or every day) user (as % of ever users)	325 (95.9)	333 (96.2)	329 (95.6)	987 (95.9)
Little cigar smoking status, No. (%)				
Never user	36 (10.6)	32 (9.3)	32 (9.3)	100 (9.7)
Ever user	303 (89.4)	314 (90.7)	312 (90.7)	929 (90.3)
Current (some days or every day) User (as % of ever users)	265 (78.2)	276 (79.8)	268 (77.9)	809 (78.6)
Nicotine dependence score (scale 1-5), mean (SD)	2.6 (1.7)	2.5 (1.7)	2.7 (1.7)	2.6 (1.7)
Frequency of seeing a warning label on a cigar package in the last 30 d (1 = never; 5 = always), mean (SD)	3.8 (1.3)	3.7 (1.3)	3.8 (1.2)	3.8 (1.2)
No. of days a daily survey was completed, mean (SD)	14.9 (4.6)	14.9 (4.7)	14.7 (4.9)	14.8 (4.7)
Primary study outcome				
Intention to quit: likelihood of quitting smoking LCCs in the next 6 mo (1 = not at all; 4 = very), mean (SD)	2.4 (1.0)	2.2 (1.0)	2.3 (1.0)	2.3 (1.0)
Secondary study outcome				
Attempted to quit smoking LCCs in the last 7 d, No. (%)	80 (23.6)	66 (19.1)	56 (16.3)	202 (19.6)

Abbreviations: FDA, US Food and Drug Administration; GED, General Educational Development; GHWs, graphic health warnings; LCCs, little cigars and cigarillos.

^a^ Includes people who reported their race as Middle Eastern or Northern African, Native Hawaiian or Other Pacific Islander, or other race, ethnicity, or origin.

^b^ FDA text-only LCC warnings: n = 345; total sample: n = 1028.

^c^ LCC GHWs: n = 338; FDA text-only LCC warnings: n = 345; no warning control: n = 342; total sample: n = 1025.

^d^ FDA text-only LCC warnings: n = 345; total sample: n = 1028.

**Figure 3.**
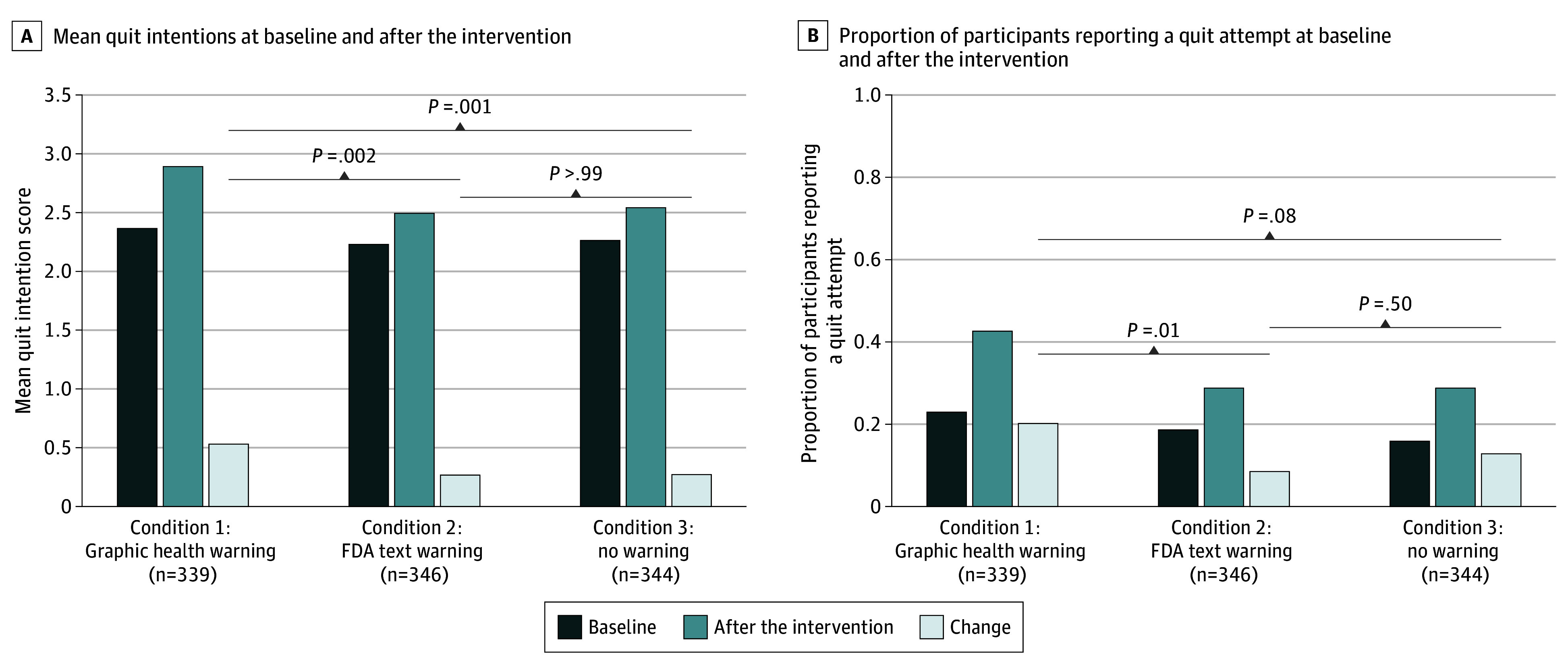
Quit Intentions and Quit Attempts at Baseline and After the Intervention and Change in Levels From Baseline to After the Intervention *P* values refer to statistical tests comparing the change in the outcomes (gray bars) among 1029 adult users of little cigars and cigarillos (LCCs) from a Qualtrics online panel who were randomized to (1) LCC graphic health warnings (n = 339), (2) existing US Food and Drug Administration (FDA) text-only warnings (n = 346), or (3) no warnings (n = 344).

Mean quit intention scores after the intervention (ie, day 21) were 2.9 (95% CI, 2.8-3.0) for LCC GHW participants, 2.5 (95% CI, 2.4-2.6) for existing FDA text-only participants, and 2.6 (95% CI, 2.4-2.7) for control participants (Figure 3A). Although quit intentions increased among participants in all groups, the change in quit intentions was significantly higher among participants assigned to the LCC GHW condition compared with the existing FDA text-only and control conditions (Figure 3A and B). Adjusting for baseline quit intentions did not substantively change the model coefficients (eTable in Supplement 2).

The mean proportion of participants reporting quit attempts after the intervention was significantly higher at 0.4 (95% CI, 0.4-0.5) for LCC GHW participants compared with 0.3 (95% CI, 0.2-0.3) for both text-only and control participants (Figure 3B). When adjusting for baseline quit attempts, the patterns of the model coefficients did not change (eTable in Supplement 2). Although quit attempts increased among participants in all groups, the increases in quit attempts were significantly higher among participants assigned to the LCC GHW condition compared with the existing FDA text-only condition (Figure 3A and B). There was no significant difference in the change in quit attempts between the LCC GHW and control groups (Figure 3B).

In each study week, participants reported butting out and forgoing LCCs between 1 and 3 times on average, with these behaviors appearing more frequently in the GHW and text-only conditions compared with controls. Participants in the LCC GHW condition were significantly more likely to report butting out both little cigars and cigarillos compared with those in the text-only condition (adjusted estimate [SE]: butting out little cigars, 0.07 [0.04]; butting out cigarillos, 0.07 [0.03]) and forgoing both little cigars and cigarillos (adjusted estimate [SE]: forgoing little cigars, 0.12 [0.03]; forgoing cigarillos, 0.13 [0.03]) compared with those in the existing FDA text-only warning condition and control condition (Table 2). The associations were not changed after adjustment for baseline behaviors or interaction between condition assignment and week.

**Table 2.** Effect of Condition Assignment on Butting Out or Forgoing Little Cigars and Cigarillos^a^

Outcome	Crude		Adjusted		Interaction by week	
	Estimate (SE)	*P* value	Estimate (SE)	*P* value	Estimate (SE)	*P* value
**Butting out little cigars**						
GHWs vs control	0.04 (0.03)	.26	0.04 (0.04)	.25	0.04 (0.04)	.26
GHWs vs text only	0.07 (0.03)	.04	0.07 (0.04)	.05	0.07 (0.04)	.05
Text only vs control	−0.031 (0.03)	.35	−0.031 (0.04)	.41	−0.030 (0.04)	.42
**Forgoing little cigars**						
GHWs vs control	0.07 (0.03)	.04	0.07 (0.03)	.04	0.07 (0.03)	.04
GHWs vs text only	0.12 (0.03)	.001	0.12 (0.03)	<.001	0.12 (0.03)	<.001
Text only vs control	−0.048 (0.03)	.17	−0.052 (0.03)	.13	−0.052 (0.3)	.13
**Butting out cigarillos**						
GHWs vs control	0.05 (0.03)	.11	0.04 (0.03)	.19	0.04 (0.03)	.21
GHWs vs text only	0.08 (0.03)	.016	0.07 (0.03)	.03	0.07 (0.03)	.04
Text only vs control	−0.026 (0.03)	.42	−0.029 (0.03)	.38	−0.028 (0.03)	.40
**Forgoing cigarillos**						
GHWs vs control	0.10 (0.03)	.002	0.10 (0.03)	.002	0.10 (0.03)	.002
GHWs vs text only	0.14 (0.03)	<.001	0.13 (0.03)	<.001	0.13 (0.03)	<.001
Text only vs control	−0.043 (0.03)	.18	−0.035 (0.03)	.27	−0.035 (0.03)	.27

Abbreviations: GHWs, graphic health warnings.

^a^ “Butting out” refers to extinguishing a little cigar or cigarillo that was already lit.

## Discussion

This study is the first, to our knowledge, to demonstrate the effectiveness of GHWs for LCCs in influencing multiple cessation behavioral outcomes. Participants exposed to our newly developed LCC GHWs in an RCT exhibited significantly greater increases in quit intentions from before to after the intervention compared with participants exposed to the text-only and control conditions, with no differences observed between the text-only and control conditions. Regarding quit attempts, the mean proportion of participants reporting quit attempts after the intervention was significantly greater in the LCC GHW condition compared with the text-only and control conditions. The change in quit attempts from before to after the intervention was also significant for LCC GHW participants compared with the text-only condition, although the change from before to after quit attempts was not significantly different between the LCC GHW and control conditions. Participants in the LCC GHW condition were also more likely to report behaviors indicative of reduced product use, such as butting out or forgoing LCCs, which are also associated with cessation behaviors.^32,33^

Our findings suggest that the existing FDA text-only warnings in the US are insufficient to influence LCC use and that LCC GHWs may play a pivotal role in reducing the appeal and consumption of LCCs among adults in the US. The fact that the FDA’s current text-only warnings had no significant effect on behavioral intentions or outcomes is not surprising, particularly because tobacco manufacturers have not been required to use them on packaging, they are not novel, and they have no graphic components. Even though these FDA warnings are not mandatory, most participants across all conditions reported having seen such a warning in the month prior to our study, suggesting widespread use of existing FDA text-only warnings by LCC manufacturers on the packaging,^18^ despite previous evidence of limited effectiveness in influencing user perceptions or behaviors.^14,15,16^ The saturation of the existing FDA text-only warnings potentially contributes to their reduced novelty or effectiveness.

The fact that the LCC GHWs increased quit intentions, quit attempts, and LCC use reduction (via butting out and foregoing) in a sample that was also highly exposed to text-only warnings in the real world emphasizes the benefit of the GHW approach in communicating the health risks of LCCs and encouraging quitting. The newly developed LCC GHWs can inform regulatory efforts in the US to provide a stronger evidentiary basis for GWHs for LCCs, helping address legal concerns about the lack of data on the specific effects of cigar warnings on cigar use and attitudes.^17^

Although our results align with prior research on cigarette GHWs, which has consistently shown their effectiveness in reducing smoking intentions, increasing quit attempts, and diminishing product appeal,^9,10^ it extends the effect of GHWs to LCCs, a major group of noncigarette tobacco products.^25^ Internationally, 138 countries have implemented GHWs on packages for cigarettes, but relatively few have taken strong actions to develop GHWs for cigars.^12^ Although a small number of countries (eg, Australia and Canada) require cigar packages to carry large GHWs, similar to those used for cigarettes,^34^ little previous evidence exists on behavioral outcomes of LCC GHWs. A recent systematic review on the effect of GHWs on tobacco products and quit intentions did not find any longitudinal interventions on cigars among the 35 reported studies, almost all of which were for cigarettes.^35^

The present study shows that the effect on LCC use may depend on the combination of new, evidence-based text statements, such as those used in our study and pictorial images that made up the LCC GHW design. The text statements that we used focused on 2 health effects within a message, and we used causal language.^23^ The pictorials depicted graphic internal and external representations of disease when possible.^21^ Such findings are consistent with the limited formative research on LCC warnings. For instance, a study of young adult cigarillo smokers showed that they paid little attention to the current FDA text-only warnings but believed that pictorial warnings would more likely discourage use among cigarillo users.^36^ In another study, pictorial warnings on cigarillo packaging also appeared to elicit greater negative emotional reactions compared with text-only warnings.^13^

Although this RCT focused on LCC GHWs delivered via an email invitation to each day’s survey, LCC GHW warnings may also show promise in affecting health outcomes for large or premium cigars. A recent experiment that used similar LCC GHWs as those used in our study on large and premium cigars sold individually at the point of sale found that the newly developed LCC GHWs were more effective than the FDA’s current text-only warnings in conveying the health risks and could increase the effectiveness of warning labels for such cigars.^37^

### Limitations

This study has several limitations. Although quit attempts are a more proximal indicator of complete smoking cessation than intention to quit,^38^ we recognized the difficulty in achieving longer-term behavior change within this brief study period vs the increased likelihood of shorter-term changes in intentions. As such, quit intentions were selected a priori as our primary outcome. Relatedly, we observed more modest increases in changes in quit attempts and butting out LCCs, such that we were not able to detect a significant difference between the GHW condition and the control condition in the magnitude of increase in quit attempts and frequency of butting out LCCs. Although a reliance on self-reported data for quit intentions and attempts could introduce bias, as participants might overreport desirable behaviors and underreport undesirable behaviors, the RCT design, with its rigorous assignment of conditions and controlled settings, helped mitigate potential confounding factors and allowed for more robust comparisons between groups. History of quit attempts was not balanced among groups after randomization, but a sensitivity analysis adjusting for baseline quit attempts did not suggest the presence of residual confounding by this variable. In addition, the short follow-up period and the use of pictorial images on cigar packages rather than a GHW on real cigar packages limits the ability to assess long-term effects, but the consistent findings across multiple measures (eg, quit intentions and reported behaviors) strengthens confidence in the immediate effects of these GHWs.

Additional research is needed examining the actual effects of GHWs on LCC packages in a natural environment, the longitudinal effects at the point of sale or other methods for implementing GHWs on large and premium cigars, and the effect of LCC GHWs on blunt use, a major avenue for the use of cigarillos.^39^ Finally, while formative work demonstrated that LCC GHWs at 30% of pack size were as good as those at 50% size,^25^ most countries internationally have adopted cigarette GHWs user warnings of 50% or more of pack size; additional research examining longitudinal effects of larger sizes of LCC GHWs is indicated.^12^

## Conclusions

This RCT fills a critical gap in tobacco control research, demonstrating for the first time the efficacy of GHWs for LCCs on longitudinally increasing quit intentions and reducing product use. These findings provide empirical evidence for regulatory bodies and policymakers to consider in advancing GHWs for LCCs.

## Notes

Supplement 1.
Trial Protocol and Statistical Analysis Plan

Supplement 2. **eFigure.** Effect of Condition on Quit Intentions (Panel A) and Quit Attempts (Panel B) at 21 Days, Adjusting for Baseline Values **eTable.** Frequency of Reported Butting Out and Forgoing LCCs in the Last 7 Days

Supplement 3.
Data Sharing Statement
