# Error in Byline and Author Contributions

**DOI:** 10.1001/jamanetworkopen.2025.35214

**Published:** 2025-09-17

In the Original Investigation titled “Little Cigar and Cigarillo Graphic Health Warnings and Quitting Behaviors: A Randomized Clinical Trial,”^1^ published August 15, 2025, there was an error in the byline and in the Author Contributions. In the byline, the seventh author’s first name was listed as Sandra and should have been listed as Sonia. In the Author Contributions, *Supervision* was listed twice, with and without Dr Enyioha’s name; the version of *Supervision* listed without Dr Enyioha’s name has been removed. This article has been corrected.^1^
