# Supplementary material for: Little Cigar and Cigarillo Graphic Health Warnings and Quitting Behaviors: A Randomized Clinical Trial
Source: JAMA Netw Open. 2025 Aug 15;8(8):e2526799. doi: 10.1001/jamanetworkopen.2025.26799 (PMC12357197; doi:10.1001/jamanetworkopen.2025.26799)
Supplement: Supplement 1. — Trial Protocol and Statistical Analysis Plan [file jamanetwopen-e2526799-s001.pdf]

Little Cigar and Cigarillo Warnings to Reduce Tobacco-Related Cancers and Disease: Randomized Controlled Trial among US Adults who use LCCs

Principal Investigator: Adam O. Goldstein, MD MPH

Version 1, 4/17/2023
Registered at ClinicalTrials.gov, ID NCT05849051, https://www.clinicaltrials.gov/ct2/show/NCT05849051

Contents

Administrative Information 4
- Trial Registration 4
- Roles and Responsibilities 4
- Funding Statement and Sponsors 5
- Abbreviations and Definitions of Terms 5

Introduction 6
- Background and Rationale 6
- Objectives and Hypotheses 6
  - Hypotheses 7
- Trial Design 7

Methods: Participants, Interventions, and Outcomes 7
- Methods Overview 7
- Participant timeline 8
- Study Setting 8
- Participants 8
  - Eligibility criteria for participation in this study: 8
- Interventions 8
  - Experimental Conditions 8
  - Warning Stimuli 9
  - Stimuli Order 11
  - Additional intervention details 13
- Outcomes 13
  - Primary 13
  - Secondary 13
  - Other 14
- Sample Size 15
- Recruitment 15
- Compensation Plan 15

Methods: Assignment of interventions 15
- Allocation and Blinding 15

Methods: Data collection management, and analysis 15
- Data collection methods 15
  - Screener 15
  - Baseline Survey: Day 0 15

Daily Surveys: Days 1-6, 8-13, and 15-20 16
Weekly Surveys: Days 7, 14 16
Post-Test Survey: Day 21 16
Data Management 16
Statistical Methods 16
Methods: Monitoring 17
Ethics and dissemination 17
Consent 17
Confidentiality 17
Declaration of Interests 18
Access to data 18
Ancillary and post-trial care 18
Dissemination Plan 18
References 19
Appendix A: Trial Consent Form 21

Administrative Information

Trial Registration

Title: Little Cigar and Cigarillo Warnings to Reduce Tobacco-Related Cancers and Disease: Randomized Controlled Trial among US Adults who use LCCs

Short Title: Little Cigar and Cigarillo Warnings

Registered at ClinicalTrials.gov, ID NCT05849051, https://www.clinicaltrials.gov/ct2/show/NCT05849051

Registration Date: May 8, 2023

Roles and Responsibilities

The research team below is responsible for the study design, management, analysis and interpretation of data; writing any reports or publications; and decision to submit any reports or publications. Participant recruitment and data collection will be completed through Qualtrics and their panel provider, with supervision by the study team.

**Name**	**Role**	**Affiliation**
Adam O. Goldstein	Principal Investigator	Department of Family Medicine, University of North Carolina at Chapel Hill<br>Lineberger Comprehensive Cancer Center, University of North Carolina at Chapel Hill
Kristen L. Jarman	Project Manager	Department of Family Medicine, University of North Carolina at Chapel Hill
Tara L. Queen	Statistician, Co-Investigator	Department of Health Behavior, Gillings School of Public Health, University of North Carolina at Chapel Hill
Sarah D. Kowitt	Co-Investigator	Department of Family Medicine, University of North Carolina at Chapel Hill<br>Lineberger Comprehensive Cancer Center, University of North Carolina at Chapel Hill
Leah M. Ranney	Co-Investigator	Department of Family Medicine, University of North Carolina at Chapel Hill<br>Lineberger Comprehensive Cancer Center, University of North Carolina at Chapel Hill
Jennifer Cornacchione Ross	Co-Investigator	Department of Health Law, Policy, and Management, Boston University, School of<br>Public Health
Paschal Sheeran	Co-Investigator	Department of Psychology and Neuroscience, University of North Carolina at Chapel Hill<br>Lineberger Comprehensive Cancer Center, University of North Carolina at Chapel Hill
James Thrasher	Co-Investigator	Department of Health Promotion, Education, and Behavior, Arnold School of Public Health, University of South Carolina
Sonia Clark	Research Staff	Department of Family Medicine, University of North Carolina at Chapel Hill
Olivia Hodgson	Graduate Research Assistant	Gillings School of Public Health, University of North Carolina at Chapel Hill

Funding Statement and Sponsors

Research reported in this publication was supported by grant number R01CA240732 from the National Cancer Institute (NCI) and the Food and Drug Administration (FDA) Center for Tobacco Products (CTP). The content is solely the responsibility of the authors and does not necessarily represent the official views of the NIH or the Food and Drug Administration.

National Cancer Institute,
National Institutes of Health
9609 Medical Center Drive
Rockville, MD 20850

Center for Tobacco Products
Food and Drug Administration
Document Control Center
10903 New Hampshire Avenue
Building 71, Room G335
Silver Spring, MD 20993-0002

Abbreviations and Definitions of Terms

Abbreviation	Definition
LCC	Little cigars and cigarillos
NIH	National Institutes of Health
NCI	National Cancer Institute
FDA	Food and Drug Administration
CTP	Center for Tobacco Products
UNC	University of North Carolina at Chapel Hill
HWL	Health warning labels
PME	Perceived message effectiveness
OTP	Other tobacco product (products other than little cigars and cigarillos)

Introduction

Background and Rationale

Over 4 million US adults regularly smoke cigars, which causes multiple cancers, including oral, esophageal, pancreatic, laryngeal, and lung cancer. (NCI ,1998; Chang, 2015) Even smoking 1-2 cigars per day is associated with elevated cancer risk. Though cigarette consumption decreased 39% from 2000 to 2015, cigar consumption increased 85%. (Wang, 2016) Of the three major types of cigars—large cigars, little cigars, and cigarillos—little cigars and cigarillos (LCC) are the most commonly used in the US. (Delnevo, Giovenco, 2017; Delnevo, Hrywna, 2017) LCC use among adults has increased, in part, because LCCs are taxed at a lower rate than cigarettes, are subject to fewer regulations and marketing restrictions, can be purchased in small pack sizes, and are exempt from flavor bans that apply to cigarettes.

In May 2016, the Food and Drug Administration (FDA) required text-only warnings on LCC packs, rotating among six statements. Research on cigarettes suggests warnings on packs should have multiple rotating sets, contain images illustrating the negative health effects associated with LCC use, and be large. (World Health Organization, 2008; Hammond, 2011; Noar, 2016) However, the evidence for cigarette warning labels cannot adequately inform implementation of improved LCC warnings for four reasons: 1) The FDA proposed cigar warnings differ from existing cigarette warnings; 2) there is no evidence on the effectiveness of the FDA proposed cigar warnings (i.e., behavioral intentions or outcomes)(Richardson, 2013; Sterling, 2013; Glasser, 2017) or evidence on efforts that might improve LCC warnings (i.e., images, larger warning size, removal of LCC flavor descriptors on packaging); 3) Courts have ruled that one type of effective tobacco warning (i.e., for cigarettes) cannot be used to justify other types of tobacco warnings, such as those for LCCs; (R.J. Reynolds v. FDA, 2012) and 4) LCC users have different demographic and consumption profiles than cigarette users (i.e., LCC users include a higher proportion of young adults (SAMHSA, 2017), and African Americans, (Nyman, 2016; SAMHSA, 2017), and LCCs are used on fewer days per month)(Nyman, 2016; Jamal, 2018), which should be taken into account when developing improved warnings.

Gaps exist in understanding which LCC warning characteristics (i.e., content, format, size) are most influential in reducing LCC use, and how additional LCC policies, such as removal of flavor descriptors on packaging, influence the impact of LCC warnings. Our project will provide new data to fill these evidence gaps.

A previous study that we conducted found that the most important aspect of warning statement text are the health effects included in the warning, and that other adjustments, for example including multiple health effects in a warning, can lead to stronger warnings (Jarman, 2021). Findings from another of our previous studies indicate that images paired with our warning statements that depict the internal harm or both internal and external harm of smoking LCCs were more effective than warnings with images that depict external harm alone (Clark, 2022). In a 2x2 experiment to assess whether warning type (warning statement + image vs. warning statement only) and warning size (30% vs. 50%) were associated with perceived message effectiveness (PME), we found that the warnings that included images were higher in terms of PME than the warnings that were text-only, and that warning size were similar in terms of PME. (Goldstein, 2023)

Given the lack of research on LCC product warnings, our overarching goal is to assess whether LCC warnings developed by the study team are more effective than the currently implemented health warnings on LCC products. Given this goal, the choice of comparators are: 1) Newly developed warnings (the six most effective warnings developed by the study team), 2) FDA proposed text-only warnings or 3) No warnings (control condition) in which participants will not see warnings.

Objectives and Hypotheses

Objectives

The proposed study will fill critical gaps regarding which characteristics make LCC warning most effective and provide needed evidence on how LCC warnings influence LCC behavioral intentions. Our overarching goal is to develop effective LCC warnings that reduce cancer and other health risks.

Hypotheses

- Newly developed warnings with images will have higher LCC quit intentions compared to FDA proposed text-only warnings and compared to the control condition in which participants do not see packs or LCC warnings
- The FDA proposed text-only warnings will have higher LCC quit intentions compared to the control condition
- The warning conditions combined will have higher LCC quit intentions compared to the control condition
- Newly developed warnings with images will have higher self-reported learning compared to FDA proposed text-only warnings

Trial Design

The study is designed to be a parallel group trial, with participants evenly allocated to each study condition, using a superiority framework.

Methods: Participants, Interventions, and Outcomes

Methods Overview

We will conduct a 3-week web-based RCT of U.S. adults who currently use LCCs. In this study, LCC warnings on packs will be electronically presented to participants over time to determine if newly developed LCC warnings increase quit intentions compared to FDA proposed text-only warnings and a control condition (in which participants do not see LCC packs or warnings.) We will apply a daily diary methodology to present LCC warnings on packs to participants over time. Qualtrics will contact, screen, consent and administer the survey. To enroll participants, Qualtrics will screen participants using our inclusion criteria and measures and invite eligible participants to enroll in the study. To collect at least 750 quality completes we anticipate enrolling up to 3,000 people.

At the beginning of the baseline survey (day 0), participants will first consent to participate in the study and then complete a questionnaire about their tobacco use and behaviors (e.g., intentions and quit attempts) and other measures of interest. At the end of the baseline questionnaire, survey software will randomly assign participants to one of the 3 study conditions. The three study conditions are: 1) Newly developed warnings (the six most effective warnings developed by the study team), 2) FDA proposed text-only warnings or 3) No warnings (control condition) in which participants will not see warnings or LCC packages. Participants will be contacted via email each day (at approximately 6am) to invite them to complete the survey for that day of the study protocol.

For subsequent days (days 1-6, 8-13, 15-20) participants will be contacted and asked to complete a daily survey which will assess their previous day use of LCCs, as well as cigarettes and e-cigarettes. During these daily surveys, participants assigned to condition 1 or 2 (i.e., the warning conditions) will view an image of a little cigar and cigarillo package with a warning according to the participant's condition. Participants within each warning condition will view a total of 6 different warnings over the course of 6 days each week, this will be repeated 3 times during the study, resulting in a total of 18 exposures. Participants will be required to view the warning for at least 5 seconds before answering questions.

On days 7 and 14 participants will be asked to complete a slightly longer survey with questions about their LCC behaviors including: number of LCCs used in the past week, butted out because they wanted to smoke less, and forgone, other tobacco use, blunt use, and quit intentions and attempts.

For the post-test on day 21, participants will be asked to complete a longer questionnaire about their current tobacco use and behaviors including current LCC smoking behavior, LCC nicotine dependence, OTP use, LCC and OTP quit intentions, and LCC and OTP quit attempts.

Participant timeline

Potential participants will be invited to complete the screener, those who are eligible will be invited to participate in the study. On Day 0, participants will be asked to complete a baseline survey, then a survey every morning for days 1-6, 8-13, and 15-20 to deliver an LCC warning (stimuli shown only for conditions 1 and 2) and assess previous day behavior. Weekly behavior surveys will be completed on days 7 and 14. There is a final post-test on day 21.

Process:

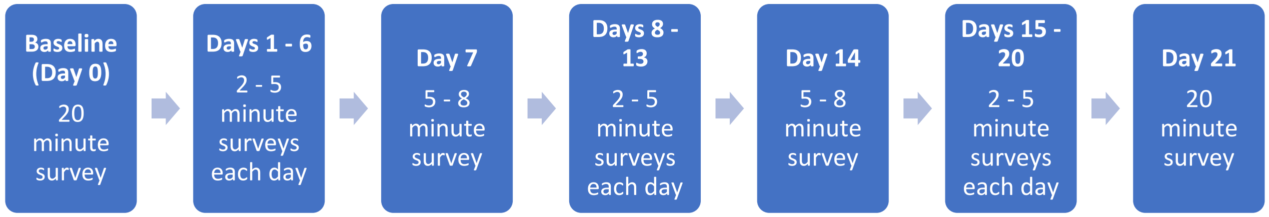

Study Setting

The study will be conducted with online surveys, so participants can complete the surveys from wherever they are. As part of eligibility criteria, all participants will reside in the United States.

Participants

Eligibility criteria for participation in this study:

1. Members of the recruitment panel (we are partnering with a panel provider for recruitment of all participants)
2. Agree to provide their honest answers
3. Current little cigar and/or cigarillo every day or some day users
4. Over 21 years old
5. Currently living in US
6. Inclusion criteria based on the design of the study:
    a. Feel comfortable taking a survey in English without help
    b. Feel comfortable taking an online survey without help
    c. Have an email address that they check regularly
    d. Have access to the internet at work or home
    e. Able to read and respond to surveys delivered to their email
    f. Able to complete 2 surveys that take approximately 20 minutes
    g. Able to complete a 5 minute survey each day for 20 days
7. Inclusion criteria based on verifying real participants:
    a. Able to verify they are not a bot using CAPTCHA
    b. Able to answer a simple, random math question

Interventions

Experimental Conditions

1. Newly developed warnings: the six most effective warnings developed in Aim 1 at 30% size
2. FDA proposed text-only warnings: the six currently proposed warnings at 30% size

3. Control group: a control condition in which participants will not see warnings or LCC packages, but will be asked to complete daily surveys on previous-day LCC behaviors

Warning Stimuli

The stimuli for conditions 1 and 2 will vary based on the day of the protocol. The packages that the study warning labels will be shown on is standardized, warning labels will be shown on both a cigarillo and little cigar package (see table below: Stimuli Shown by Condition), and the packages will be purple and branded with the fictitious name “Brentfield” which has successfully been used in prior research to minimize the influence of brand loyalty and pre-existing brand perceptions. The warnings will be shown on the bottom 30% of the front of the package. Warning text for both warning conditions will be centered and the marker word “WARNING” will be placed on a separate line. In accordance with FDA guidelines, warning text will be displayed in an arial bold type font. Across all warnings, the font in little cigar warnings was size 12 and in cigarillo packages, the font was size 14. (FDA, 2020)

For the two warning conditions, LCC packages will be centered and placed on either a plain white background or light grey, blurred background. When present, background images will be consistent with locations where LCCs could be purchased (i.e., at a counter or in a grocery store, or blank). The background is used to give context to the stimuli that may help participants focus on it and give a more real world feeling.

Stimuli Shown by Condition:

Condition	Six Warning Messages Presented	Example LCC package depicted on each background		
		Transparent (White)	Grocery Store	Cigar Counter
Newly developed warnings (Condition 1)	WARNING: Cigar smoking causes pharyngeal and throat cancer.	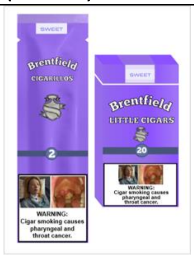	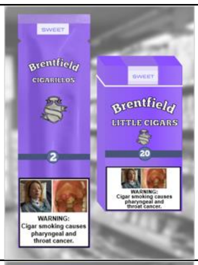	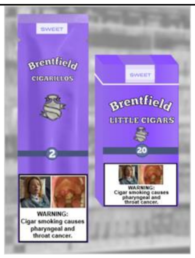
	WARNING: Cigar smoking causes stroke and blood clots.	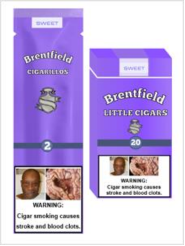	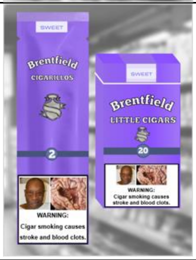	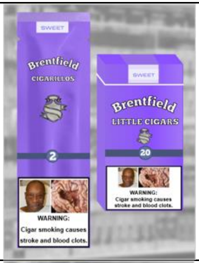
	WARNING: Cigar smoking causes colon cancer.	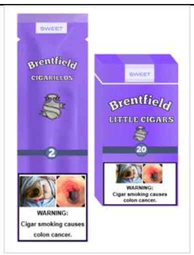	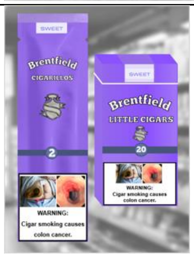	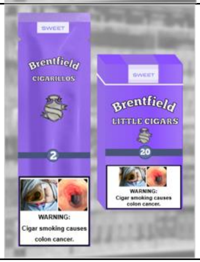

	WARNING: Cigar smoking causes bladder cancer and blood in urine.	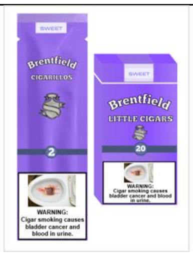	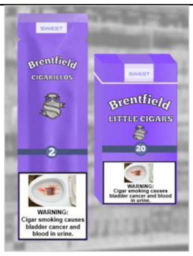	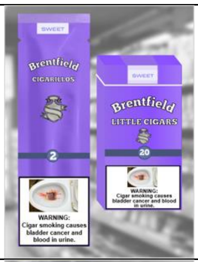
	WARNING: Cigar smoking causes esophageal cancer.	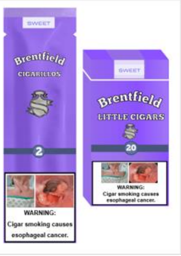	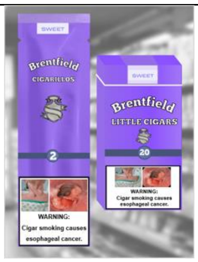	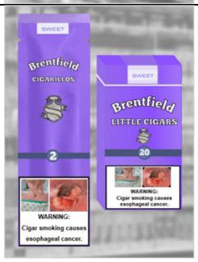
	WARNING: Cigar smoking causes lung cancer and lung disease.	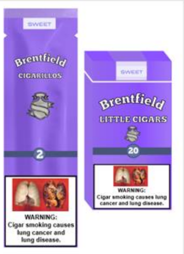	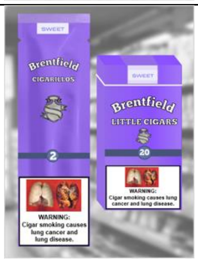	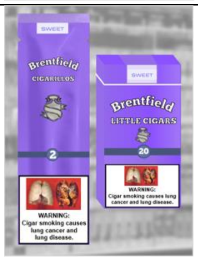
FDA proposed text-only warnings (Condition 2)	WARNING: Cigar smoking can cause cancers of the mouth and throat, even if you do not inhale.	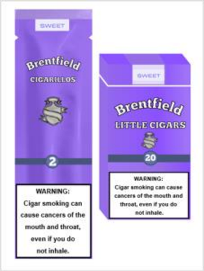	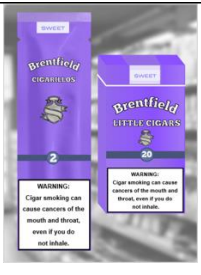	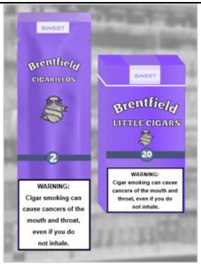
	WARNING: Cigar smoking can cause lung cancer and heart disease.	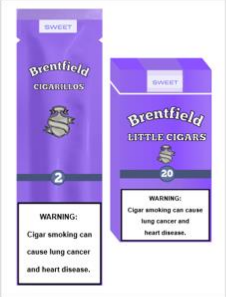	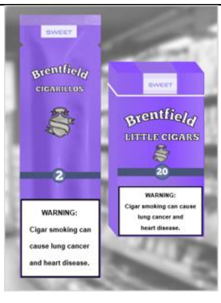	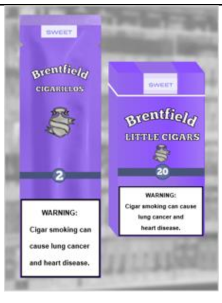
	WARNING: Cigars are not a safe alternative to cigarettes.	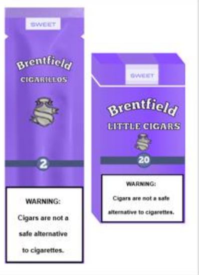	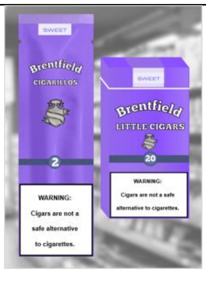	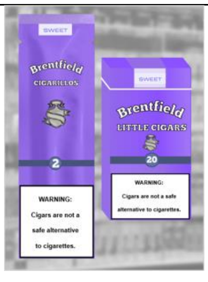

	WARNING: Tobacco smoke increases the risk of lung cancer and heart disease, even in nonsmokers.	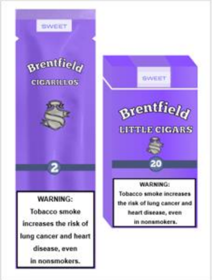	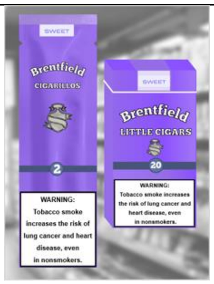	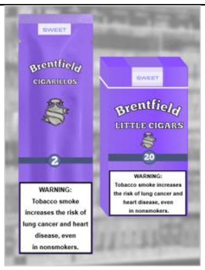
	WARNING: Cigar use while pregnant can harm you and your baby.	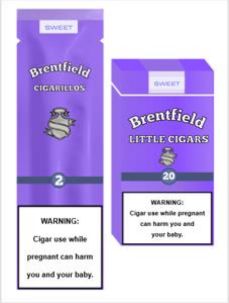	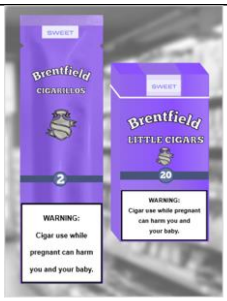	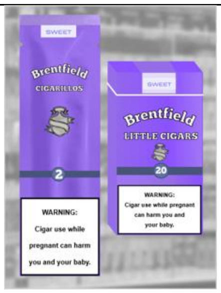
	WARNING: This product contains nicotine. Nicotine is an addictive chemical.	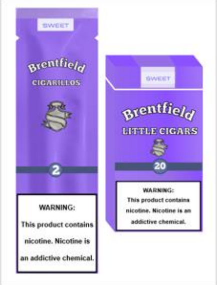	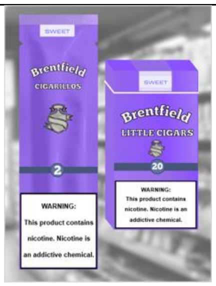	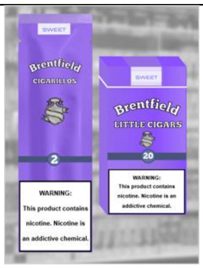
No stimuli: Control (Condition 3)	For condition 3, the control, participants were not presented any stimuli.	N/A	N/A	N/A

Stimuli Order

Day	Message	Condition 1	Condition 2	Background
Day 1	A	Throat	Mouth	Plain
Day 2	B	Lung	Heart	Grocery
Day 3	F	Stroke	Nicotine	Counter
Day 4	C	Colon	Unsafe	Plain
Day 5	E	Bladder	Baby	Counter
Day 6	D	Esoph	SHS	Grocery
Day 8	B	Lung	Heart	Plain
Day 9	C	Colon	Unsafe	Grocery
Day 10	A	Throat	Mouth	Counter
Day 11	D	Esoph	SHS	Counter
Day 12	F	Stroke	Nicotine	Grocery
Day 13	E	Bladder	Baby	Plain
Day 15	C	Colon	Unsafe	Counter
Day 16	D	Esoph	SHS	Plain
Day 17	B	Lung	Heart	Counter
Day 18	E	Bladder	Baby	Grocery
Day 19	A	Throat	Mouth	Grocery
Day 20	F	Stroke	Nicotine	Plain

Condition 3 does not have a stimuli order because that condition does not see packages or warnings, their daily surveys are only to assess behavior

Message Order (based on Latin Square design)

- In week 1, participants see the following message order: A-B-F-C-E-D (sequence 1)
- In week 2, participants see the following message order: B-C-A-D-F-E (sequence 2)
- In week 3, participants see the following message order: C-D-B-E-A-F (sequence 3)

Message by Condition:

Message Letter	Condition 1: Text + Image Warning	Condition 2: FDA Text Only Warning
A	WARNING: Cigar smoking causes pharyngeal and throat cancer.<br>(Throat1)	WARNING: Cigar smoking can cause cancers of the mouth and throat, even if you do not inhale.<br>(Mouth2)
B	WARNING: Cigar smoking causes lung cancer and lung disease.<br>(Lung1)	WARNING: Cigar smoking can cause lung cancer and heart disease.<br>(Heart2)
C	WARNING: Cigar smoking causes colon cancer.<br>(Colon1)	WARNING: Cigars are not a safe alternative to cigarettes.<br>(Unsafe2)
D	WARNING: Cigar smoking causes esophageal cancer.<br>(Esoph1)	WARNING: Tobacco smoke increases the risk of lung cancer and heart disease, even in nonsmokers.<br>(SHS2)
E	WARNING: Cigar smoking causes bladder cancer and blood in urine.<br>(Bladder1)	WARNING: Cigar use while pregnant can harm you and your baby.<br>(Baby2)

Message Letter	Condition 1: Text + Image Warning	Condition 2: FDA Text Only Warning
F	WARNING: Cigar smoking causes stroke and blood clots. (Stroke1)	WARNING: This product contains nicotine. Nicotine is an addictive chemical. (Nicotine2)

Background Order (based on Latin Square Design)

We will have 3 background options (A=Plain, B=Counter, C=Grocery), they will be counterbalanced in order based on day

- Message A will have background sequence: A, B, C (Sequence 1)
- Message B will have background sequence: C, A, B (Sequence 3)
- Message C will have background sequence: A, C, B (Sequence 2)
- Message D will have background sequence: C, B, A (Sequence 4)
- Message E will have background sequence: B, A, C (Sequence 6)
- Message F will have background sequence: B, C, A (Sequence 5)

Additional intervention details

Participants can withdraw from the study if they choose by no longer responding to the survey links and may withdraw their data by contacting the UNC study team.

Multiple strategies have been implemented to maximize adherence to the trial protocol, including reaching out to participants via email with invitations and reminders each day, compensating them based on completion of the most important questionnaires.

There is no relevant concomitant care or interventions that are permitted or prohibited during the trial.

Outcomes

Primary

- LCC quit intentions at post test (day 21)
    - Average quit intention score measured by survey, Quit intention measured with 3 questions, the final quit intention score is a mean of the response to the 3 questions, on a scale of 1 to 4, where 1 indicates low intention to quit, and 4 indicates a high intention to quit.

Secondary

- Number of LCCs smoked in past day (each day)
    - Measured daily by survey
- Number of days smoked LCCs in past week (days 7, 14, and 21)
    - Measured weekly by survey
- Number of LCCs smoked in the past week (days 7, 14, and 21)
    - Measured weekly by survey
- Butting out LCCs (days 7, 14, and 21)
    - Measured weekly by survey
- Forgoing LCCs (days 7, 14, and 21)
    - Measured weekly by survey
- LCC quit attempts (days 7, 14, and 21)
    - Measured weekly by survey
- LCC quit intentions (days 7 and 14)

    - Average quit intention score measured by survey, Quit intention measured with 3 questions, the final quit intention score is a mean of the response to the 3 questions, on a scale of 1 to 4, where 1 indicates low intention to quit, and 4 indicates a high intention to quit.
- Self-reported learning (day 21) (only conditions 1 and 2)
    - Measured by survey. Self-reported learning measured with one item on a 1 to 5 scale, where 1 indicates no learning, and 5 indicates a great deal of learning from the warnings in the study.

Other

- Past week large cigars (days 7, 14, and 21)
    - Measured weekly by survey
- Past week cigarettes (days 7, 14, and 21)
    - Measured weekly by survey
- Past week e-cigarettes (days 7, 14, and 21)
    - Measured weekly by survey
- Past week smokeless (days 7, 14, and 21)
    - Measured weekly by survey
- Past week e-cigarettes (days 7, 14, and 21)
    - Measured weekly by survey
- Past week hookah (days 7, 14, and 21)
    - Measured weekly by survey
- Past week oral nicotine (days 7, 14, and 21)
    - Measured weekly by survey
- Past week something else (days 7, 14, and 21)
    - Measured weekly by survey
- Past week blunt use (y/n) (days 7, 14, and 21)
    - Measured weekly by survey
- Number of blunts used in last week (days 7, 14, and 21)
    - Measured weekly by survey
- Motivation to quit smoking LCCs (day 21)
    - Measured by survey with 1 question, where 1 indicates no motivation to quit smoking LCCs and 7 indicates a very high motivation to quit smoking LCCs
- Tripartite risk (day 21)
    - Mean tripartite risk, Measured by survey with 3 questions on a scale of 1 to 4, where 1 indicates a low risk perception of LCCs and 4 indicates a high risk perception of LCCs
- Reactance to warnings (day 21) (only conditions 1 and 2)
    - Mean reactance to warnings, measured by survey with 3 questions on a scale of 1 to 5 where 1 indicates low reactance to warnings and 5 indicates a high reactance to warnings
- Warning recall (day 21) (only conditions 1 and 2)
    - Measured by survey. Warning recall measured with one item, where 1 indicates that they do remember the warnings from the study and 0 indicates that they do not remember the warnings in the study.
- Warning recognition (day 21) (only conditions 1 and 2)
    - Measured by survey with 6 questions
- Perceptions of recent LCCs smoked (days 7, 14, and 21)
    - Measured by survey with 3 questions
- Conversations about the warnings (days 7, 14, and 21) (only conditions 1 and 2)
    - Measured by survey with 2 questions
- Nicotine dependence (5 items) (day 21)
    - Measured by survey with 5 questions.
    -

Sample Size

The sample size is based on quit intentions effect sizes from an RCT comparing graphic cigarette warnings to text only warnings (Brewer, 2016). Based on the effect size observed in that study, we would have 80% power at $\alpha=0.05$ to detect a small change in differences between our groups ($f=0.12$).

Recruitment

We are working with Qualtrics research services to conduct this study and enroll participants from one of the panel providers that they work with. Qualtrics has access to high quality research panels across the US. They are able to leverage these panels to provide timely and reliable data collection to UNC and other research universities. We have high confidence that Qualtrics will be able to meet our recruitment goals.

Compensation Plan

Qualtrics will manage participant incentives. Participants in studies conducted by Qualtrics receive an incentive based on the length of the survey and their specific profile. The types of rewards vary and may include cash, airline miles, gift cards, redeemable points, sweepstakes entrance and vouchers. Participants who complete only the screening survey will receive a small incentive. For this study, participants who complete the baseline, 6 of the daily message surveys, at least one of the weekly surveys (day 7 or day 14), and day 21 will receive an incentive. Participants who complete only a few of the surveys but do not satisfy the criteria above will not receive an incentive.

Methods: Assignment of interventions

Allocation and Blinding

Participants will not be informed specifically about the possible interventions that they may be assigned to. Researchers will not be blinded to the condition that participants had been assigned to, however all outcome measures will be assessed via online survey. At the end of the baseline survey, survey software will randomly assign participants to one of the three study arms. Participants will have an equal chance of being randomized to each study arm.

Methods: Data collection management, and analysis

Data collection methods

Data collection will be conducted using Qualtrics web survey platform, questionnaires and references for measures can be found in the study measures document. Where possible, the measures used are previously developed and validated measures. Multiple strategies have been implemented to maximize adherence to the trial protocol, including reaching out to participants via email with invitations and reminders each day, and compensating them based on completion of the most important questionnaires.

Screener

Qualtrics will invite potential participants to complete the screening survey and people who are eligible will be invited to complete the baseline survey. The screener is used to assess participant eligibility and collect demographic data.

Baseline Survey: Day 0

- Eligible participants will be invited to participate in the study and complete the baseline survey to enroll
- The first part of the baseline survey will be a consent form with an agreement to participate in the study
- The baseline survey contains baseline tobacco use questions (e.g., motivation to quit, previous year LCC quit attempts, nicotine dependence, etc.).
- At the end of the baseline questionnaire, survey software will randomly assign participants to one of the 3 study conditions

Daily Surveys: Days 1-6, 8-13, and 15-20

- There are 18 daily surveys, which align with each participant's condition. Participants in conditions 1 and 2 will be shown stimuli (in accordance with their assigned condition) and condition 3 will not see any stimuli. Participants will stay in their condition throughout the study. Depending on the day, participants will see a different message
- Daily surveys will be sent out at 6am EST and will close at 2am EST (gives west coast until 11pm to complete surveys). There will be a reminder email sent in the afternoon.

Weekly Surveys: Days 7, 14

- Weekly behavior surveys will include questions about LCC behaviors including: number of LCCs used in the past week, butted out because they wanted to smoke less, and forgone), other tobacco use (OTP), blunt use, and quit intentions and attempts.)
- The weekly surveys will be sent out at 6am EST and will close 48 hours later. There will be a reminder email sent in the afternoon, and the next day.

Post-Test Survey: Day 21

- For the post-test on day 21, participants will be asked to complete a longer questionnaire about their current tobacco use and behaviors including current LCC smoking behavior, LCC nicotine dependence, OTP use, LCC and OTP quit intentions, and LCC and OTP quit attempts.
- Participants in conditions 1 and 2 will be asked recognition questions to assess whether they can recognize warnings shown to them in their condition
- Participants will also be asked about their study experience
- The post-test surveys will be sent out at 6am EST and will close 48 hours later. There will be a reminder email sent in the afternoon, and the next day.

Data Management

Data management will be conducted by the study team. Multiple processes have been implemented to ensure high data quality, including internal pilots to test the protocol and ensure that data collection instruments are working properly, and ongoing data monitoring will be conducted during data collection to ensure that any issues with data quality are caught early and fixed. A pilot of the protocol with actual participants will be conducted before the full study is launched and participants will be asked about their experience in the study at the end of the pilot to further ensure that the protocol is working as intended and ensure a high quality data collection.

Statistical Methods

We will analyze the data using intention-to-treat analyses. Our primary outcome is LCC quit intention at post-test. We will model LCC quit intentions with message condition (warnings with images, FDA proposed text-only warnings, and a no warning control) as the between-participant predictor.

Analysis methods will depend on the time point that the outcome was measured. Post-test measures will be analyzed differently than repeated measures from the daily survey and weekly surveys, as outlined below. All models will include a categorical indicator of day to control for the durable linear effect of time.

Analysis methods for post-test measures will use linear regression for continuous outcomes, including the primary outcome of quit intentions, and logistic regression for dichotomous outcomes. Imputation will be used so that all participants who were randomized to an intervention and completed at least one daily survey will be included in the analysis, consistent with intention to treat practices.

Secondary models will examine differences between groups for items measured multiple times in either daily or weekly surveys (ex., LCC behavior measures). For these models, we will use linear mixed modeling to account for the repeated

measure design. Mixed models are appropriate to use and robust to missing data, so we will be able to include everyone who completed at least 1 daily questionnaire, consistent with intention to treat practices.

Secondary models will also be run for the primary outcome as well as secondary outcomes that include the number of daily surveys completed (This is a measure that is equivalent to the dose of the stimuli for the active condition and also applies to the control condition)

LCC behavior is collected separately for cigarillos and little cigars and will be combined in the main analyses for these outcomes (ex. Number of cigarillos in past day and number of little cigars in past day combined for number of LCCs in past day). We also plan to run separate analyses for cigarillo behavior and little cigar behavior.

Additional information about the statistical analysis can be found in the Statistical Analysis Plan.

Methods: Monitoring

To ensure maximum protection of human subjects, we submitted our research project information to the School of Medicine Data Safety and Monitoring Board at the University of North Carolina at Chapel Hill and asked them to make the final decision regarding risk to participants and whether or not our studies should receive their supervision. A representative of the board agreed that given that the study involves a low risk behavioral intervention in a healthy population, that oversight by the board was not necessary.

Adverse events are not expected in this trial due to the minimal risks to participants, and no threat to participant health, so plans for collecting, assessing and reporting adverse events are not necessary.

We have no plans for auditing trial conduct.

Ethics and dissemination

This trial was granted an exemption by UNC's IRB under IRB # 22-2531.

All protocol changes will be communicated to the study team, and any change in participant interaction will be approved by UNC's IRB. Protocol changes will be tracked in an appendix table with new versions after initial approval including the date, version number, and a summary of changes.

Consent

Consent will be collected electronically as part of the baseline survey at the beginning of the study. At the beginning of the baseline survey, participants will view the consent form with an agreement to participate in the study. Participants will only be able to proceed with the study if they agree to participate in the study.

Confidentiality

We will take the following steps to minimize the risk to a breach of confidentiality via unauthorized access, use, disclosure, modification, loss or theft of participants' information: We will use and enforce appropriate security measures including physical, technical and administrative safeguards. Data will be stored on a university secure server that is password accessed and is only accessible to key research personnel. All study personnel are required to have valid training on ethics of research on human subjects and to complete confidentiality certification procedures upon employment. The research team will not have access to personal identifying information.

This study proposes research that has been determined to include Security Level 1 data security requirements. The PI has agreed to accept responsibility for managing these risks appropriately in consultation with departmental and/or campus security personnel. The Data Security Requirements addendum can be reviewed here: https://guides.lib.unc.edu/datasecurity/irbis

Declaration of Interests

The study team declare that they have no competing interests.

Access to data

We will make data collection instruments available for public use after data collection and analyses are complete. On request, we will also make data sets available, stripped of individual identifiers, following publication of the relevant study's main findings. We may submit deidentified data collected in this study to a data repository, such as UNC Odum Institute's Dataverse (https://dataverse.unc.edu/dataverse/unc) to make our deidentified data publicly available. As part of submitting the data to a repository we will provide basic information about how the data was collected and upload a deidentified dataset to the repository.

Ancillary and post-trial care

This trial presents no more than minimal risk to participants, does not collect data on harms to participants or assess interim data during the trial. There is no need for ancillary or post-trail care.

Dissemination Plan

The study team plans to disseminate study findings via conference presentation and peer reviewed manuscripts in scientific journals. The study team will also share findings via ClinicalTrials.gov registration. We will follow authorship guidelines depending on the journal to which manuscripts are submitted. There are currently no plans to make a participant level dataset public.

References

Brewer NT, Hall MG, Noar SM, et al. Effect of pictorial cigarette pack warnings on changes in smoking behavior: A randomized clinical trial. JAMA internal medicine. 2016;176(7):905-912.

Chang CM, Corey CG, Rostron BL, Apelberg BJ. Systematic review of cigar smoking and all cause and smoking related mortality. BMC Public Health. Apr 24 2015;15:390.

Clark SA, Kowitt SD, Lazard A, Jarman KL, Sheeran P, Ranney LM., Cornacchione Ross J, Kistler CE, Thrasher JF, Goldstein AO. Identifying effective images for cigar warnings. Oral presentation at the 2022 Annual Meeting of the Society for Research on Nicotine and Tobacco.

Delnevo CD, Giovenco DP, Miller Lo EJ. Changes in the Mass-merchandise Cigar Market since the Tobacco Control Act. Tobacco regulatory science. 2017;3(2):8-16.

Delnevo CD, Hrywna M, Giovenco DP, Miller Lo EJ, O'Connor RJ. Close, but no cigar: certain cigars are pseudo-cigarettes designed to evade regulation. Tob Control. May 2017;26(3):349-354.

Food and Drug Administration. Cigar Labeling and Warning Statement Requirements. 2020. Available at: https://www.fda.gov/tobacco-products/labeling-and-warning-statements-tobacco-products/cigar-labeling-and-warning-statement-requirements.

Glasser AM, Johnson AL, Rose SW, et al. Correlates of cigar use by type and flavor among US young adults: 2011-2015. Tob Regul Sci. 2017;3(2):59-71.

Goldstein AO, Kowitt SD, Jarman KL, Clark SA, Cornacchione Ross J, Sheeran P, Enyioha C, Queen T, Ranney LM, Thrasher JF. The Impact of Graphic Imagery and Warning Size on the Effectiveness of Cigar Warnings. Poster presented at the 2023 Annual Meeting of the Society for Research on Nicotine and Tobacco.

Hammond D. Health warning messages on tobacco products: a review. Tob Control. Sep 2011;20(5):327-337.

Jamal A, Phillips E, Gentzke AS, et al. Current Cigarette Smoking Among Adults - United States, 2016. MMWR Morb Mortal Wkly Rep. Jan 19 2018;67(2):53-59.

Jarman KL, Kistler C, Thrasher J, Kowitt SD, Ranney LM, Cornacchione Ross J, Sheeran P, Lazard A, Goldstein AO. Designing effective warning text for cigars: a discrete choice experiment among adult cigar smokers. Oral presentation at the 2021 NIH Tobacco Regulatory Science Meeting.

National Cancer Institute, DHHS, NIH. Cigars, Health Effects and Trends, Smoking and Tobacco Control Monograph No. 9. Bethesda, MD: U.S. Department of Health and Human Services, Public Health Service, National Institutes of Health, National Cancer Institute;1998. NIH Publication No. 98-430.

Noar SM, Hall MG, Francis DB, Ribisl KM, Pepper JK, Brewer NT. Pictorial cigarette pack warnings: a metaanalysis of experimental studies. Tob Control. May 2016;25(3):341-354.

Nyman AL, Sterling KL, Weaver SR, Majeed BA, Eriksen MP. Little Cigars and Cigarillos: Users, Perceptions, and Reasons for Use. Tob Regul Sci. Jul 2016;2(3):239-251.

R.J. Reynolds Tobacco Co. et al. v. U.S. Food & Drug Admininistration., U.S. District Court, District of Columbia, No. 11-01482. 2012.

Richardson A, Rath J, Ganz O, Xiao H, Vallone D. Primary and dual users of little cigars/cigarillos and large cigars: demographic and tobacco use profiles. Nicotine Tob Res. 2013:ntt053.

Sterling K, Berg CJ, Thomas AN, Glantz SA, Ahluwalia JS. Factors associated with small cigar use among college students. Am J Health Behav. May 2013;37(3):325-333.

Substance Abuse and Mental Health Services Administration, Center for Behavioral Health Statistics and Quality. Results from the 2016 national survey on drug use and health: detailed tables. 2017. Available at: https://www.samhsa.gov/data/sites/default/files/NSDUH-DetTabs-2016/NSDUH-DetTabs-2016.pdf. Accessed September 25, 2018.

Wang TW, Kenemer B, Tynan MA, Singh T, King B. Consumption of combustible and smokeless tobacco - United States, 2000-2015. MMWR Morb Mortal Wkly Rep. Dec 09 2016;65(48):1357-1363.

World Health Organization. WHO Framework Convention on Tobacco Control: Elaboration of Guidelines for Implementation of Article 11 of the Convention. 2008. Available at: http://apps.who.int/gb/fctc/PDF/cop3/FCTC_COP3_7-en.pdf. Accessed 06/11/2017.

Protocol structure and contents based on SPIRIT 2013 Statement: Defining standard protocol items for clinical trials, https://www.spirit-statement.org/wp-content/uploads/2013/01/SPIRIT-Checklist-download-8Jan13.pdf

Appendix A: Trial Consent Form

University of North Carolina at Chapel Hill
Research Information Sheet
IRB Study #: 22-2531
Principal Investigator: Adam Goldstein, MD MPH

The purpose of this research study is to study tobacco use over a 3 week period of time. You are being asked to take part in this research study because you are an adult that uses little cigars or cigarillos.

Being in a research study is completely voluntary. You can choose not to be in this research study. You can also say yes now and change your mind later.

If you agree to take part in this research, you will be asked to complete a survey today, and then be asked to complete a survey every day for the next 3 weeks. Your participation in this study will take about 1.5 to 3 hours spread over today and the next 3 weeks.

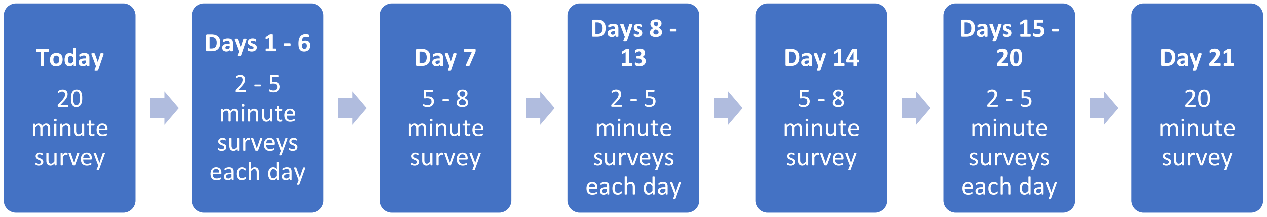

We expect that 3,000 people will take part in this research study.

You can choose not to answer any question you do not wish to answer. You can also choose to stop taking the survey at any time. You must be at least 21 years old to participate. If you are younger than 21 years old, please stop now.

The possible risks to you in taking part in this research are:

- Statements or questions in the survey may make you feel uncomfortable or evoke emotional reactions. We anticipate this risk to be minimal and manageable.
- Survey questions may ask you about your opinions or behaviors that you would prefer to keep private. If you do not feel comfortable answering a question, you may skip it
- There is a risk of loss of confidentiality of the data, we will minimize these risks by using and enforcing appropriate data protection standards.

To protect your identity as a research subject, the research data will not be stored with your name, and the researcher(s) will not share your information with anyone. In any publication about this research, your name or other private information will not be used.

Most people outside the research team will not see your name on your research information. This includes people who try to get your information using a court order in the United States. One exception is if you agree that we can give out research information with your name on it or for research projects that have been approved under applicable rules. Other exceptions are for information that is required to be reported under law, such as information about child or disabled abuse or neglect or certain harmful diseases that can be spread from one

person to another. Personnel of a government agency sponsoring the study may also be provided information about your involvement in the research study.

The de-identified data collected as part of this study may be used for additional research or included as part of a study data registry without additional consent.

If you complete this study, you will be given an incentive from the panel provider. To complete this study, you must complete the survey today, the surveys on day 7, 14, and on or within 48 hours after day 21, and at least 6 of the surveys on the other days.

If you have any questions about this research, please contact the study team by calling (919)966-3016 or emailing JKristen@email.unc.edu. If you have questions or concerns about your rights as a research subject, you may contact the UNC Institutional Review Board at 919-966-3113 or by email to IRB_subjects@unc.edu.

Do you agree to participate?
[ ] Yes, I agree to participate

Appendix B. List of Measures by Timeframe

Screener	Baseline	Daily (with messages)	Day 7 and 14 - **Weekly Behavior Assessment**	Post
Quality Participants<br>• Quality Control<br>• Captcha<br>• Simple Math Problem<br>Assess LCC use<br>• Ever Cigarillo<br>• Days per month smoked cigarillo<br>• Every/some days cigarillo<br>• Cigarillo brand<br>• Ever little cigar<br>• Days per month smoked little cigar<br>• Every/some days little cigar<br>• Little cigar brand<br>Demographics<br>• Age<br>• Hispanic/Latino origin<br>• Race<br>• Gender<br>• State<br>Other inclusion criteria:<br>• Comfort with survey in English<br>• Comfort with survey on computer<br>• Email access<br>• Access to internet<br>• Ability to complete emailed surveys	Consent Form<br>**Weekly Behavior Assessment**<br>Other tobacco use questions (14-28 items)<br>• Cigarette use (2 items)<br>• Motivation to quit smoking<br>• Previous year LCC quit attempts<br>• Nicotine Dependence (5 items)<br>• Tripartite Risk (3 items)<br>• Self-Efficacy<br>• Response Efficacy<br>• Open Mindedness<br>• OTP Quit Intentions (0-7 items)<br>• OTP Quit Attempts (0-7 items)<br>Additional Demographics<br>• Sexual orientation<br>• Education<br>• Household size<br>• Income<br>• Subjective Financial Status<br>• Zip code	• Past day cigarillos<br>• Past day little cigars<br>• Past day cigarettes<br>• Past day e-cigarettes<br>• Warning/Study Stimuli<br>• Attention to stimuli	LCC Behavior (5 – 9 items)<br>• Memory Cue<br>• Past week Cigarillos<br>• Days smoked<br>• Number smoked<br>• Butting out cigarillos<br>• Forgoing cigarillos<br>• Past week little cigars<br>• Days smoked<br>• Number smoked<br>• Butting out little cigars<br>• Forgoing little cigars<br>OTP (2-9 items)<br>• Tobacco products used in past week<br>• Past week large cigars<br>• Past week cigarettes<br>• Past week smokeless<br>• Past week e-cigarettes<br>• Past week hookah<br>• Past week oral nicotine<br>• Past week something else<br>• Past week NRT<br>Past day items (4 items)<br>• Past day cigarillos<br>• Past day little cigars<br>• Past day cigarettes<br>• Past day e-cigarettes<br>Intentions, etc. (14 items)	**Weekly Behavior Assessment**<br>Other tobacco use questions (12-26 items)<br>• Motivation to quit smoking<br>• Nicotine Dependence (5 items)<br>• Tripartite Risk (3 items)<br>• Self-Efficacy<br>• Response Efficacy<br>• OTP Quit Intentions (0-7 items)<br>• OTP Quit Attempts (0-7 items)<br>Stimuli Responses (11 items)<br>• Reactance (3 items)<br>• Self-reported learning<br>• Recall<br>• Recognition (6 items)<br>Quality Control Questions (8-11 items)<br>• See stimuli (+follow up)<br>• Read text (+follow up)<br>• Understand questions (+follow up)<br>• Do study again<br>• Recommend study to friend<br>• Annoyed by study<br>• Anything to share

• Ability to complete longer surveys<br>• Ability to complete daily surveys			• Past week blunts (2 items)<br>• LCC quit intentions (3 items)<br>• LCC Quit attempts<br>• Conversations (2 items)<br>• Other LCC warnings<br>• Cognitive elaboration (2 items)<br>• Perceptions of recent LCCs smoked (3 items)	• Difficulty with behavior recall
Between 18 and 22 items – estimate about 5 – 6 minutes	Between 20 and 34 items + consent, + 25-36 from weekly behavior assessment.<br><br>Estimate it will take 14 – 20 minutes when combined with screener and weekly behavior assessment	3 items or stimuli and 5 items – estimate about 2 minutes to complete	Between 25 and 36 items – estimate about 5 – 8 min	Between 31 and 48 items, plus 25-36 from weekly behavior assessment.<br><br>Estimate it will take 15 – 22 minutes when combined with weekly behavior assessment

Little Cigar and Cigarillo Warnings to Reduce Tobacco-Related Cancers and Disease: Randomized Controlled Trial among US Adults who use LCCs

Statistical Analysis Plan

Contents

Administration Information 3
ClinicalTrials.gov ID: 3
Study Protocol Version 3
SAP Revision History 3
Roles and Responsibilities 3
Introduction 3
Background and Rationale 3
Objectives 4
Study Methods 4
Trial Design 4
Randomization 4
Sample Size 4
Framework 5
Statistical Interim Analyses and Stopping Guidance 5
Timing of Final Analysis 5
Timing of Outcome Assessments 6
Statistical Principles 8
Confidence Intervals and P Values 8
Adherence and Protocol Deviations 8
Analysis Populations 8
Trial Population 9
Screening Data 9
Eligibility 9
Recruitment 10
Withdrawal/follow-up 11
Baseline participant characteristics 11
Analysis 12
Outcome definitions 12
Analysis Methods 14
Harms 16
Statistical Software 17
References 17

Administration Information

ClinicalTrials.gov ID: NCT05849051, https://www.clinicaltrials.gov/ct2/show/NCT05849051

Study Protocol Version LCC Adult RCT Protocol V1

SAP Revision History

Version Number	Date	Summary of Changes
1	May 23, 2023	SAP Created, and agreed to

Roles and Responsibilities

Name	Affiliation	Role
Kristen L. Jarman, MSPH	UNC Family Medicine	Project Manager, Document Originator
Olivia Hodgson	UNC Gillings School of Public Health, UNC Family Medicine	Graduate Research Assistant
Tara L. Queen, PhD	UNC Gillings School of Public Health, Department of Health Behavior	Co-Investigator, Statistical Analyst
Sarah D. Kowitt, PhD	UNC Family Medicine	Co-Investigator
Adam O. Goldstein, MD MPH	UNC Family Medicine	Project PI

Introduction

Background and Rationale

Over 4 million US adults regularly smoke cigars, which causes multiple cancers, including oral, esophageal, pancreatic, laryngeal, and lung cancer.[1,2] Even smoking 1-2 cigars per day is associated with elevated cancer risk. Though cigarette consumption decreased 39% from 2000 to 2015, cigar consumption increased 85%.[3] Of the three major types of cigars—large cigars, little cigars, and cigarillos—little cigars and cigarillos (LCC) are the most commonly used in the US.[4,5] LCC use among adults has increased, in part, because LCCs are taxed at a lower rate than cigarettes, are subject to fewer regulations and marketing restrictions, can be purchased in small pack sizes, and are exempt from flavor bans that apply to cigarettes.

In May 2016, the Food and Drug Administration (FDA) required text-only warnings on LCC packs, rotating among six statements. Research on cigarettes suggests warnings on packs should have multiple rotating sets, contain images illustrating the negative health effects associated with LCC use, and be large.[6–8] However, the evidence for cigarette warning labels cannot adequately inform implementation of improved LCC warnings for four reasons: 1) The FDA proposed cigar warnings differ from existing cigarette warnings; 2) there is no evidence on the effectiveness of the FDA proposed cigar warnings (i.e., behavioral intentions or outcomes)[9–11] or evidence on efforts that might improve LCC warnings (i.e., images, larger warning size, removal of LCC flavor descriptors on packaging); 3) Courts have ruled that one type of effective tobacco warning (i.e., for cigarettes) cannot be used to justify other types of tobacco warnings, such as those for LCCs;[12] and 4) LCC users have different demographic and consumption profiles than cigarette users (i.e., LCC users include a higher proportion of young adults,[13] and African Americans,[13,14] and LCCs are used on fewer days per month),[14,15] which should be taken into account when developing improved warnings.

Gaps exist in understanding which LCC warning characteristics (i.e., content, format, size) are most influential in reducing LCC use, and how additional LCC policies, such as removal of flavor descriptors on packaging, influence the impact of LCC warnings. Our project will provide new data to fill these evidence gaps.

Our research on LCC warnings has found that the health effects included in warnings are the most important aspect of warning the warning statement text, and including multiple warnings can lead to stronger warnings,[16] that images paired with our warning statements that depict the internal harm or both internal and external harm of smoking LCCs were more effective.[17] In a 2x2 experiment to assess whether warning type (warning statement + image vs. warning statement only) and warning size (30% vs. 50%) were associated with perceived message effectiveness (PME), we found that the warnings that included images were higher in terms of PME than the warnings that were text-only, and that warning size were similar in terms of PME.[18]

Given the lack of research on LCC product warnings, our overarching goal is assess whether LCC warnings developed by the study team are more effective than the currently implemented health warnings on LCC products. Given this goal, the choice of comparators are: 1) Newly developed warnings (the six most effective warnings developed by the study team), 2) FDA proposed text-only warnings or 3) No warnings (control condition) in which participants will not see warnings.

Objectives

The proposed study will fill critical gaps regarding which characteristics make LCC warning most effective and provide needed evidence on how LCC warnings influence LCC behavioral intentions. Our overarching goal is to develop effective LCC warnings that reduce cancer and other health risks.

Hypotheses

- Newly developed warnings with images will have higher LCC quit intentions compared to FDA proposed text-only warnings and compared to the control condition in which participants do not see packs or LCC warnings
- The FDA proposed text-only warnings will have higher LCC quit intentions compared to the control condition
- The warning conditions combined will have higher LCC quit intentions compared to the control condition
- Newly developed warnings with images will have higher self-reported learning compared to FDA proposed text-only warnings

Study Methods

Trial Design

Three group parallel trial.

The three conditions are:

1. Newly developed warnings: the six most effective warnings developed in Aim 1 at 30% size
2. FDA proposed text-only warnings: the six currently proposed warnings at 30% size
3. Control group: a control condition in which participants will not see warnings, but will be asked to complete daily surveys on previous-day LCC behaviors

Randomization

At the end of the baseline survey, survey software will randomly assign participants to one of the three study arms. Participants will have an equal chance of being randomized to each study arm.

Sample Size

We will analyze the data using intention-to-treat analyses. Our primary outcome is LCC quit intentions at post test (day 21). We will model LCC quit intentions with experimental condition as the between-participant predictor. *We hypothesize that our most effective warnings from Aim 1 will increase LCC quit intentions compared to existing, FDA-proposed warnings and a control condition.*

The 750 good complete sample size for our Aim 2 RCT is based on quit intentions effect sizes from an RCT comparing graphic cigarette warnings to text only warnings.[19] Based on the effect size observed in that study, we would have 80% power at $\alpha=0.05$ to detect a small change in differences between our groups (f=0.12).

Framework
Superiority – standard hypothesis testing framework

Statistical Interim Analyses and Stopping Guidance
NA, this study is no more than minimal risk to participants and does not pose a risk to their health, so we did not carry out any interim analyses or set guidelines for stopping the trial early.

Timing of Final Analysis
Final analysis will take place after all data collection for the RCT is complete.

Timing of Outcome Assessments

Screener	Baseline	Daily (with messages)	Day 7 and 14 - **Weekly Behavior Assessment**	Post
Quality Participants<br>• Quality Control<br>• Captcha<br>• Simple Math Problem<br>Assess LCC use<br>• Ever Cigarillo<br>• Days per month smoked cigarillo<br>• Every/some days cigarillo<br>• Cigarillo brand<br>• Ever little cigar<br>• Days per month smoked little cigar<br>• Every/some days little cigar<br>• Little cigar brand<br>Demographics<br>• Age<br>• Hispanic/Latino origin<br>• Race<br>• Gender<br>• State<br>Other inclusion criteria:<br>• Comfort with survey in English<br>• Comfort with survey on computer<br>• Email access<br>• Access to internet<br>• Ability to complete emailed surveys<br>• Ability to complete longer surveys	Consent Form<br>**Weekly Behavior Assessment**<br>Other tobacco use questions (14-28 items)<br>• Cigarette use (2 items)<br>• Motivation to quit smoking<br>• Previous year LCC quit attempts<br>• Nicotine Dependence (5 items)<br>• Tripartite Risk (3 items)<br>• Self-Efficacy<br>• Response Efficacy<br>• Open Mindedness<br>• OTP Quit Intentions (0-7 items)<br>• OTP Quit Attempts (0-7 items)<br>Additional Demographics<br>• Sexual orientation<br>• Education<br>• Household size<br>• Income<br>• Subjective Financial Status<br>• Zip code	• Past day cigarillos<br>• Past day little cigars<br>• Past day cigarettes<br>• Past day e-cigarettes<br>• Warning/Study Stimuli<br>• Attention to stimuli	LCC Behavior (5 – 9 items)<br>• Memory Cue<br>• Past week Cigarillos<br>• Days smoked<br>• Number smoked<br>• Butting out cigarillos<br>• Forgoing cigarillos<br>• Past week little cigars<br>• Days smoked<br>• Number smoked<br>• Butting out little cigars<br>• Forgoing little cigars<br>OTP (2-9 items)<br>• Tobacco products used in past week<br>• Past week large cigars<br>• Past week cigarettes<br>• Past week smokeless<br>• Past week e-cigarettes<br>• Past week hookah<br>• Past week oral nicotine<br>• Past week something else<br>• Past week NRT<br>Past day items (4 items)<br>• Past day cigarillos<br>• Past day little cigars<br>• Past day cigarettes<br>• Past day e-cigarettes<br>Intentions, etc. (14 items)<br>• Past week blunts (2 items)	**Weekly Behavior Assessment**<br>Other tobacco use questions (12-26 items)<br>• Motivation to quit smoking<br>• Nicotine Dependence (5 items)<br>• Tripartite Risk (3 items)<br>• Self-Efficacy<br>• Response Efficacy<br>• OTP Quit Intentions (0-7 items)<br>• OTP Quit Attempts (0-7 items)<br>Stimuli Responses (11 items)<br>• Reactance (3 items)<br>• Self-reported learning<br>• Recall<br>• Recognition (6 items)<br>Quality Control Questions (8-11 items)<br>• See stimuli (+follow up)<br>• Read text (+follow up)<br>• Understand questions (+follow up)<br>• Do study again<br>• Recommend study to friend<br>• Annoyed by study<br>• Anything to share<br>• Difficulty with behavior recall

• Ability to complete daily surveys			• LCC quit intentions (3 items)<br>• LCC Quit attempts<br>• Conversations (2 items)<br>• Other LCC warnings<br>• Cognitive elaboration (2 items)<br>• Perceptions of recent LCCs smoked (3 items)	

Statistical Principles

Confidence Intervals and P Values

Level of statistical significance

α=0.05

Description and rationale for any adjustment for multiplicity and, if so detailing how the type 1 error is to be controlled

NA

Confidence Intervals to be reported

95% CI

Adherence and Protocol Deviations

Definition of adherence to the intervention and how this is assessed including extent of exposure

Compliance with the protocol is assessed as follows:

- Completion of daily surveys, during which participants view intervention stimuli. % Compliance = number of daily surveys completed / 18 (number of surveys supposed to be completed)*100%
- Completion of the weekly behavior survey at days 7 and 14
- Completion of post survey
- To be considered 'complete' for incentive purposes, the participant must complete the baseline, at least 6 daily surveys, at least one of the surveys at day 7 and 14, and the post survey at day 21

Description of how adherence to the intervention will be presented

- Adherence for the daily surveys will be presented with completion % as well as the mean number complete by treatment group
- Completion of weekly behavior surveys at days 7 and 14 will be presented by % completing each by treatment group
- Completion of post test surveys will be presented by % completing each by treatment group

Definition of protocol deviations for the trial

- Minor protocol deviation: change in eligibility between screening and baseline
- Minor protocol deviation: completing the wrong sequence of message days due to a survey programming error
- Major protocol deviation: No protocol deviations in this trial will impact the safety or physical or mental integrity of the participants in the trial. Therefore, we do not classify any protocol deviations as 'major'

Description of which protocol deviations will be summarized

- Protocol deviations will not be summarized overall, but data related to the deviations will be included in manuscripts. For example, we will not include how many people completed an extra 'day' of the protocol, but we will report the mean number of days participants completed by treatment group.

Analysis Populations

Definition of analysis populations

The primary outcome analysis of quit intentions at post test will be analyzed using multiple imputation such that everyone who was randomized to one of the study conditions will be included in the analysis, and those with missing outcome data will have their outcomes imputed.

For outcomes that were measured daily, all participants that completed at least one of the daily surveys will be included in the planned repeated measure models.

To analyze outcomes from the weekly surveys, all participants that completed at least one of the weekly surveys (including the post test) will be included in the planned repeated measure models.

Trial Population

Screening Data

Screening data will be provided based on the CONSORT guidelines, and CONSORT flow chart will be included with publications.

Eligibility

Minimum Age: 21 Years
Maximum Age: NA
Sex: All
Gender Based: No
Inclusion criteria:

1. Members of the recruitment panel (we are partnering with a panel provider for recruitment of all participants)
2. Agree to provide their honest answers
3. Current little cigar and/or cigarillo every day or some day users
4. Over 21 years old
5. Currently living in US
6. Inclusion criteria based on the design of the study:
    a. Feel comfortable taking a survey in English without help
    b. Feel comfortable taking an online survey without help
    c. Have an email address that they check regularly
    d. Have access to the internet at work or home
    e. Able to read and respond to surveys delivered to their email
    f. Able to complete 2 surveys that take approximately 20 minutes
    g. Able to complete a 5 minute survey each day for 20 days
7. Inclusion criteria based on verifying real participants:
    a. Able to verify they are not a bot using CAPTCHA
    b. Able to answer a simple, random math question

Recruitment

Recruitment data will be provided based on the CONSORT guidelines. The following CONSORT chart will be used as a template for reporting recruitment.

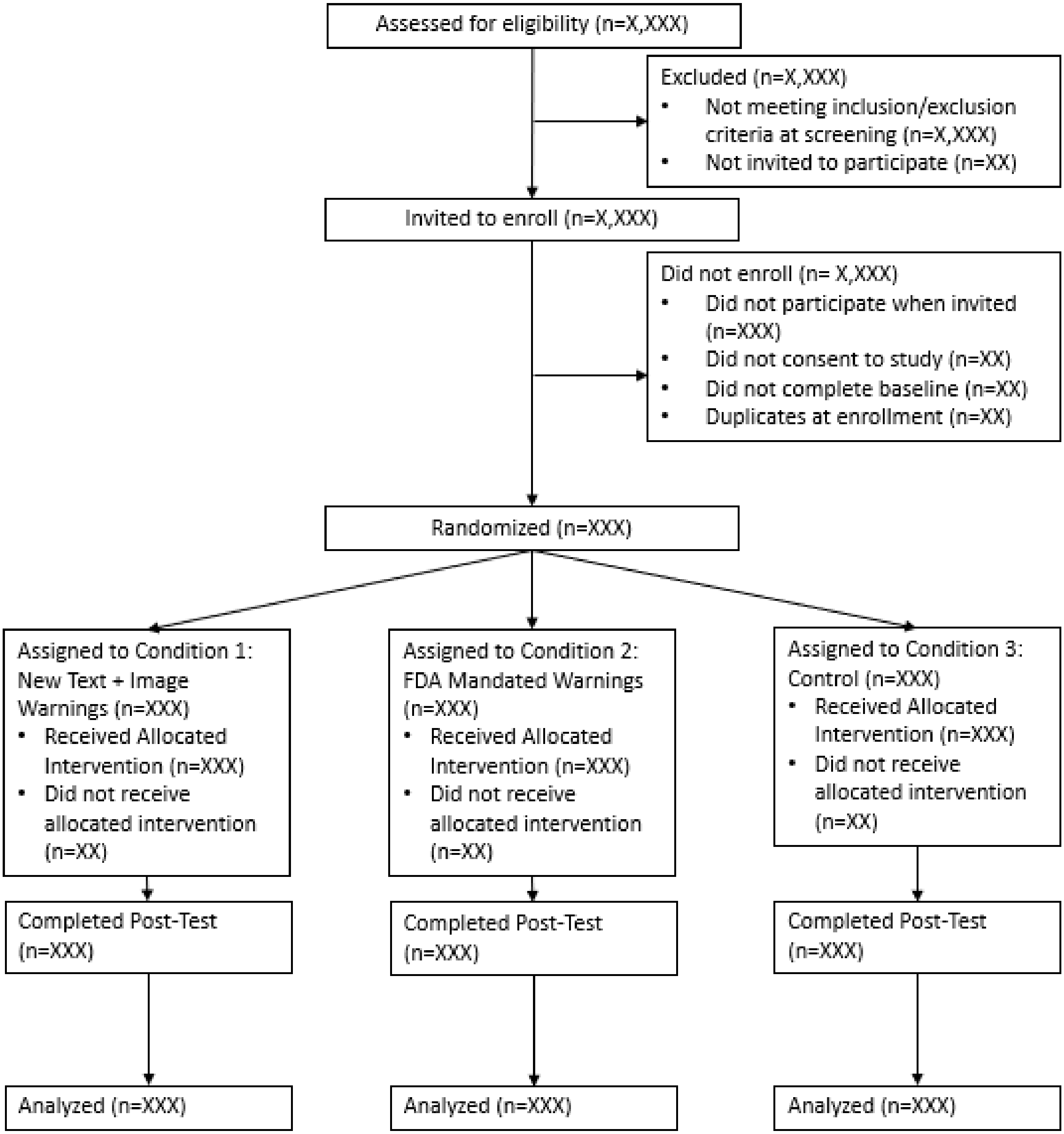

To update consort flow chart see 'CONSORT Flow Chart V1.pptx' in Protocol folder

Withdrawal/follow-up

Level of withdrawal

- Data collection and the intervention occur using the same surveys, when a participant withdraws from data collection, they are withdrawing from follow up and the intervention.

Timing of withdrawal/lost to follow-up data

- Timing of withdrawal will be presented by treatment group, and categorized into each of the following timepoints:
    - After baseline but before beginning intervention (before completing first daily survey)
    - During intervention (days 1-20)
    - During or after post-test follow up period (day 21 or after)

Reasons and details of how withdrawal/lost to follow-up data will be presented

- Numbers (with reasons) of loss to follow up and withdrawal over the course of the trial will be summarized by treatment group

Baseline participant characteristics

List of baseline characteristics to be summarized

See Draft Table 1 below.

Details of how baseline characteristics will be descriptively summarized

Baseline characteristics will be descriptively summarized as show in Draft Table 1.

Draft Table 1. Baseline Sample Characteristics

Sample Characteristics	Condition 1 (n=XXX) % or mean	Condition 2 (n=XXX) % or mean	Condition 3 (n=XXX) % or mean	Total Sample (n=XXX) % or mean
Gender				
Male				
Female				
Other/Non-binary				
Age, years				
Race				
White				
Black or African American				
American Indian or Alaska Native				
Asian				
Middle Eastern or Northern African				
Native Hawaiian or other Pacific Islander				
Some other race, ethnicity or origin				
Ethnicity				
Latino/Hispanic				
Non-Latino/Hispanic				
Education				
<High School (HS)				

G12 or GED, HS Diploma				
Some College				
Associate's degree				
Bachelor's degree				
Graduate or professional degree				
Poverty Status				
Below Poverty Line				
Above Poverty Line				
Sexual Orientation				
Straight or heterosexual				
Gay, lesbian, or bisexual				
Cigarillo Smoking Status				
Never user				
Ever User				
Current (Some day or every day) User				
Little Cigar Smoking Status				
Never user				
Ever User				
Current (Some day or every day) User				
Nicotine Dependence Score				
Number of Times Participants Viewed a Warning During Follow Up (number of daily surveys completed for control condition)				

Analysis

Outcome definitions

Primary

- LCC quit intentions at post test (day 21)
    - Average quit intention score measured by survey, Quit intention measured with 3 questions, the final quit intention score is a mean of the response to the 3 questions, on a scale of 1 to 4, where 1 indicates low intention to quit, and 4 indicates a high intention to quit.

Secondary

- Number of LCCs smoked in past day (each day)
    - Measured daily by survey
- Number of days smoked LCCs in past week (days 7, 14, and 21)
    - Measured weekly by survey
- Number of LCCs smoked in the past week (days 7, 14, and 21)
    - Measured weekly by survey
- Butting out LCCs (days 7, 14, and 21)
    - Measured weekly by survey
- Forgoing LCCs (days 7, 14, and 21)
    - Measured weekly by survey

- LCC quit attempts (days 7, 14, and 21)
    - Measured weekly by survey
- LCC quit intentions (days 7 and 14)
    - Average quit intention score measured by survey, Quit intention measured with 3 questions, the final quit intention score is a mean of the response to the 3 questions, on a scale of 1 to 4, where 1 indicates low intention to quit, and 4 indicates a high intention to quit.
- Self-reported learning (day 21) (only conditions 1 and 2)
    - Measured by survey. Self-reported learning measured with one item on a 1 to 5 scale, where 1 indicates no learning, and 5 indicates a great deal of learning from the warnings in the study.

Other

- Past week large cigars (days 7, 14, and 21)
    - Measured weekly by survey
- Past week cigarettes (days 7, 14, and 21)
    - Measured weekly by survey
- Past week e-cigarettes (days 7, 14, and 21)
    - Measured weekly by survey
- Past week smokeless (days 7, 14, and 21)
    - Measured weekly by survey
- Past week e-cigarettes (days 7, 14, and 21)
    - Measured weekly by survey
- Past week hookah (days 7, 14, and 21)
    - Measured weekly by survey
- Past week oral nicotine (days 7, 14, and 21)
    - Measured weekly by survey
- Past week something else (days 7, 14, and 21)
    - Measured weekly by survey
- Past week blunt use (y/n) (days 7, 14, and 21)
    - Measured weekly by survey
- Number of blunts used in last week (days 7, 14, and 21)
    - Measured weekly by survey
- Motivation to quit smoking LCCs (day 21)
    - Measured by survey with 1 question, where 1 indicates no motivation to quit smoking LCCs and 7 indicates a very high motivation to quit smoking LCCs
- Tripartite risk (day 21)
    - Mean tripartite risk, Measured by survey with 3 questions on a scale of 1 to 4, where 1 indicates a low risk perception of LCCs and 4 indicates a high risk perception of LCCs
- Reactance to warnings (day 21) (only conditions 1 and 2)
    - Mean reactance to warnings, measured by survey with 3 questions on a scale of 1 to 5 where 1 indicates low reactance to warnings and 5 indicates a high reactance to warnings
- Warning recall (day 21) (only conditions 1 and 2)
    - Measured by survey. Warning recall measured with one item, where 1 indicates that they do remember the warnings from the study and 0 indicates that they do not remember the warnings in the study.
- Warning recognition (day 21) (only conditions 1 and 2)
    - Measured by survey with 6 questions
- Perceptions of recent LCCs smoked (days 7, 14, and 21)
    - Measured by survey with 3 questions
- Conversations about the warnings (days 7, 14, and 21) (only conditions 1 and 2)
    - Measured by survey with 2 questions
- Nicotine dependence (5 items) (day 21)
    - Measured by survey with 5 questions.

Analysis Methods

Analysis methods will depend on the time point that the outcome was measured. Post test measures will be analyzed differently than measures from the daily surveys, as outlined below.

Primary Outcome – Quit Intentions at Post Test

What analysis method will be used and how treatment effects will be presented

Linear regression for continuous outcomes

Any adjustment for covariates

Covariates included in the analysis model (in addition to treatment group):

- Baseline quit intentions

Imputation Details:

- Covariates included for the imputation (in addition to treatment group):
    - Daily surveys completed (number of days out of 18 that a daily survey was completed)
    - Baseline and weekly measures for that outcome
    - Gender (Male, Female, Other) [20]
    - Age (continuous)
    - Poverty Status (Above or Below Poverty Line)[21,22]
    - Education (6 categories)
    - Nicotine Dependence Score [23]
- The imputation will be separate by treatment group[24]

Methods used for assumptions to be checked for statistical methods

For continuous outcomes, we assume the distribution of dependent variable given a predictor variable (intervention) is 1) independent 2) normality 3) constant variance (homogeneity of variance). We can check normality with Q-Q plot of the residual or perform Wilk-Shapiro test and use a scatter plot of residual on predictor variable (intervention) to check independence and constant variance.

Details of alternative methods to be used if distributional assumptions do not hold (eg normality, proportional hazards, etc)

If we have a violation for normality assumption, we can have two options: 1) transform our data so that the shape of our response variable become normally distributed or 2) choose the non-parametric method like Kruskal-Wallis which does not require the assumption of normality. The ANOVA can be considered a robust test against the normality assumption though. If the assumption of homogeneity of variances has been violated, we can use Welch or Brown and Forsythe test. Even though we alternatively perform a Kruskal-Wallis test, in most cases, Welch test is best. We don't expect to have dependency issues in our data.

Any planned sensitivity analyses for each outcome where applicable

We will do several sensitivity analyses to ensure …

1. A sensitivity analysis using a 'complete case analysis' framework – where we include only people that completed the baseline, at least 1 weekly assessment, post, and at least 6 daily assessments
2. A sensitivity analysis that includes only participants that completed at least 1 daily survey – these are just the people that received the assigned intervention
3. A sensitivity analysis that includes dose as a covariate in the model
4. Due to limitations in survey programming, participants are able to open and complete the daily survey more than one time, although they have no incentive to do so. A sensitivity analysis will examine whether including the number of times a person completed a daily survey yields a meaningfully different result than the number of days that a survey was completed.

Any planned subgroup analyses for each outcome including how subgroups are defined

We will conduct subgroup analyses with young adults as well as with participants who are African American and/or Black.

Missing Data

Multiple imputation will be used so that everyone who was randomized to an intervention will be included in the primary analysis

Additional Analyses

None a priori

Daily Questionnaire Measures

What analysis method will be used and how treatment effects will be presented

Mixed models to account for repeated measures of the same participant.

Any adjustment for covariates

Covariates included in the analysis model (in addition to treatment group):

- Baseline measure of the outcome of interest

Methods used for assumptions to be checked for statistical methods

Linear mixed model is an extremely flexible for modeling continuous outcomes and are robust to violations of some of their assumptions. The following are the assumptions for linear mixed model: 1) Independent (The outcomes of different people are assumed to be statistically independent of each other.) Note: the outcomes within a person are correlated. 2) Normality (random effects are assumed to be normally distributed with mean zero and the residuals are normally distributed.)

Details of alternative methods to be used if distributional assumptions do not hold (eg normality, proportional hazards, etc)

Because mixed models are robust to violations of some of their assumptions, we do not anticipate needing to use an alternative method.

Any planned sensitivity analyses for each outcome where applicable

None a priori

Any planned subgroup analyses for each outcome including how subgroups are defined

We will conduct subgroup analyses with young adults as well as with participants who are African American and/or Black.

Missing Data

Mixed models are appropriate to use and robust to missing data, so that everyone who was randomized to an intervention will be included in the analysis

Additional Analyses

None a priori

Weekly Questionnaire Measures (Including questions measured weekly and in post)

What analysis method will be used and how treatment effects will be presented

Mixed models to account for repeated measures of the same participant.

Any adjustment for covariates

Covariates included in the analysis model (in addition to treatment group):

- Baseline measure of the outcome of interest

Methods used for assumptions to be checked for statistical methods

Linear mixed model is an extremely flexible for modeling continuous outcomes and are robust to violations of some of their assumptions. The following are the assumptions for linear mixed model: 1) Independent (The outcomes of different people are assumed to be statistically independent of each other.) Note: the outcomes within a person are correlated. 2) Normality (random effects are assumed to be normally distributed with mean zero and the residuals are normally distributed.)

Details of alternative methods to be used if distributional assumptions do not hold (eg normality, proportional hazards, etc)

Because mixed models are robust to violations of some of their assumptions, we do not anticipate needing to use an alternative method.

Any planned sensitivity analyses for each outcome where applicable

None a priori

Any planned subgroup analyses for each outcome including how subgroups are defined

We will conduct subgroup analyses with young adults as well as with participants who are African American and/or Black.

Missing Data

Mixed models are appropriate to use and robust to missing data so that everyone who was randomized to an intervention will be included in the primary analysis

Additional Analyses

None a priori

Post Questionnaire Only Measures

What analysis method will be used and how treatment effects will be presented

Regression for linear outcomes, logistic for binary and categorical outcomes

Any adjustment for covariates

Covariates included in the analysis model (in addition to treatment group):

- Baseline measure of the outcome of interest (NA if the measure is not included in the baseline)

Methods used for assumptions to be checked for statistical methods

Linear mixed model is an extremely flexible for modeling continuous outcomes and are robust to violations of some of their assumptions. The following are the assumptions for linear mixed model: 1) Independent (The outcomes of different people are assumed to be statistically independent of each other.) Note: the outcomes within a person are correlated. 2) Normality (random effects are assumed to be normally distributed with mean zero and the residuals are normally distributed.)

Details of alternative methods to be used if distributional assumptions do not hold (eg normality, proportional hazards, etc)

Because mixed models are robust to violations of some of their assumptions, we do not anticipate needing to use an alternative method.

Any planned sensitivity analyses for each outcome where applicable

None a priori

Any planned subgroup analyses for each outcome including how subgroups are defined

We will conduct subgroup analyses with young adults as well as with participants who are African American and/or Black.

Missing Data

Mixed models are appropriate to use and robust to missing data so that everyone who was randomized to an intervention will be included in the primary analysis

Additional Analyses

None a priori

Harms

Adverse events were not expected in this trial due to the minimal risks to participants, and no threat to participant health, so this section is not applicable.

Statistical Software

Statistical analyses will be conducted in SAS 9.4 (Cary, NC)

References

Final Dataset: filename, location

Data Cleaning Syntax: filename, location

Study Protocol: RCT_Protocol_V5, \\ad.unc.edu\med\fammed\Projects\Goldstein Projects\Tobacco\TCORS\Aim 3 RCT\Protocol

Analysis plan compiled according to "Guidelines for the Content of Statistical Analysis Plans in Clinical Trials" [25]

1. National Cancer Institute. *Cigars, Health Effects and Trends, Smoking and Tobacco Control Monograph No. 9*.; 1998.
2. Chang CM, Corey CG, Rostron BL, Apelberg BJ. Systematic review of cigar smoking and all cause and smoking related mortality. *BMC Public Health*. 2015;15(1):390. doi:10.1186/s12889-015-1617-5
3. Wang TW, Kenemer B, Tynan MA, Singh T, King B. Consumption of Combustible and Smokeless Tobacco - United States, 2000-2015. *MMWR Morb Mortal Wkly Rep*. 2016;65(48):1357-1363. doi:10.15585/mmwr.mm6548a1
4. Delnevo CD, Giovenco DP, Miller Lo EJ. Changes in the Mass-merchandise Cigar Market since the Tobacco Control Act. *Tob Regul Sci*. 2017;3(2 Suppl 1):S8-S16.
5. Delnevo CD, Hrywna M, Giovenco DP, Miller Lo EJ, O'Connor RJ. Close, but no cigar: certain cigars are pseudo-cigarettes designed to evade regulation. *Tob Control*. 2017;26(3):349-354. doi:10.1136/tobaccocontrol-2016-052935
6. *Conference of the Parties to the WHO Framework Convention on Tobacco Control Elaboration of Guidelines for Implementation of Article 11 of the Convention*.; 2008.
7. Hammond D. Health warning messages on tobacco products: a review. *Tob Control*. 2011;20(5):327-337. doi:10.1136/tc.2010.037630
8. Noar SM, Hall MG, Francis DB, Ribisl KM, Pepper JK, Brewer NT. Pictorial cigarette pack warnings: a meta-analysis of experimental studies. *Tob Control*. 2016;25(3):341-354. doi:10.1136/tobaccocontrol-2014-051978
9. Richardson A, Rath J, Ganz O, Xiao H, Vallone D. Primary and Dual Users of Little Cigars/Cigarillos and Large Cigars: Demographic and Tobacco Use Profiles. *Nicotine & Tobacco Research*. 2013;15(10):1729-1736. doi:10.1093/ntr/ntt053
10. Sterling K, Berg CJ, Thomas AN, Glantz SA, Ahluwalia JS. Factors associated with small cigar use among college students. *Am J Health Behav*. 2013;37(3):325-333. doi:10.5993/AJHB.37.3.5
11. Glasser AM, Johnson AL, Rose SW, et al. Correlates of Cigar Use by Type and Flavor among US Young Adults: 2011-2015. *Tob Regul Sci*. 2017;3(2):59-71. doi:10.18001/TRS.3.2(Suppl1).7
12. United States Court of Appeals FOR THE DISTRICT OF COLUMBIA CIRCUIT. *R.J. Reynolds Tobacco Company, et al., Appellees v. Food and Drug Administration, et al., Appellants*.(2012). Accessed July 4, 2018. https://www.gpo.gov/fdsys/pkg/USCOURTS-caDC-12-05063/pdf/USCOURTS-caDC-12-05063-0.pdf
13. Substance Abuse and Mental Health Services Administration C for BHS and Q. *RESULTS FROM THE 2016 NATIONAL SURVEY ON DRUG USE AND HEALTH: DETAILED TABLES*.; 2017. Accessed September 18, 2019. https://www.samhsa.gov/data/sites/default/files/NSDUH-DetTabs-2016/NSDUH-DetTabs-2016.pdf
14. Nyman AL, Sterling KL, Weaver SR, Majeed BA, Eriksen MP. Little cigars and cigarillos: users, perceptions, and reasons for use. *Tob Regul Sci*. 2016;2(3):239-251.
15. Jamal A, Phillips E, Gentzke AS, et al. Current Cigarette Smoking Among Adults — United States, 2016. *MMWR Morb Mortal Wkly Rep*. 2018;67(2):53-59. doi:10.15585/mmwr.mm6702a1

16. Jarman KL, Kistler CE, Thrasher JF, et al. Identifying attributes of effective cigar warnings: a choice-based conjoint experiment in an online survey of US adults who smoke cigars. *BMJ Open*. 2024;14(12):e088525. doi:10.1136/bmjopen-2024-088525
17. Clark SA, Kowitt SD, Jarman KL, et al. The Role of Harm Visibility for Pictorial Health Warning Labels on Cigars. *Nicotine and Tobacco Research*. 2025;27(4):714-721. doi:10.1093/ntr/ntae113
18. Goldstein AO, Yang MZ, Jarman KL, et al. Impact of warning pictorials and size on perceived effectiveness of cigar warning labels in a nationally representative between-subjects experiment. *BMJ Open*. 2025;15(1):e088482. doi:10.1136/bmjopen-2024-088482
19. Brewer NT, Hall MG, Noar SM, et al. Effect of Pictorial Cigarette Pack Warnings on Changes in Smoking Behavior: A Randomized Clinical Trial. *JAMA Intern Med*. 2016;176(7):905-912. doi:10.1001/jamainternmed.2016.2621
20. The GenIUSS Group. *Best Practices for Asking Questions to Idenfity Transgender and Other Gender Minority Respondents on Population-Based Surveys*.; 2014. Accessed June 21, 2018. https://williamsinstitute.law.ucla.edu/wp-content/uploads/geniuss-report-sep-2014.pdf
21. Department of Health and Human Services. *2017 Annual Update of the HHS Poverty Guidelines*. Vol 82.; 2017.
22. Brewer NT, Jeong M, Mendel JR, et al. Cigarette pack messages about toxic chemicals: a randomised clinical trial. *Tob Control*. Published online April 13, 2018:tobaccocontrol-2017-054112. doi:10.1136/tobaccocontrol-2017-054112
23. Sung HY, Wang Y, Yao T, Lightwood J, Max W. Polytobacco Use and Nicotine Dependence Symptoms Among US Adults, 2012–2014. *Nicotine & Tobacco Research*. 2018;20(Suppl 1):S88. doi:10.1093/NTR/NTY050
24. Sullivan TR, White IR, Salter AB, Ryan P, Lee KJ. Should multiple imputation be the method of choice for handling missing data in randomized trials? *Stat Methods Med Res*. 2018;27(9):2610-2626. doi:10.1177/0962280216683570
25. Gamble C, Krishan A, Stocken D, et al. Guidelines for the Content of Statistical Analysis Plans in Clinical Trials. *JAMA*. 2017;318(23):2337. doi:10.1001/jama.2017.18556
