# Supplementary material for: Little Cigar and Cigarillo Graphic Health Warnings and Quitting Behaviors: A Randomized Clinical Trial
Source: JAMA Netw Open. 2025 Aug 15;8(8):e2526799. doi: 10.1001/jamanetworkopen.2025.26799 (PMC12357197; doi:10.1001/jamanetworkopen.2025.26799)
Supplement: Supplement 2. — eFigure. Effect of Condition on Quit Intentions (Panel A) and Quit Attempts (Panel B) at 21 Days, Adjusting for Baseline Values eTable. Frequency of Reported Butting Out and Forgoing LCCs in the Last 7 Days [file jamanetwopen-e2526799-s002.pdf]

Supplementary Online Content

Goldstein AO, Jarman KL, Ranney LM, et al. Little cigar and cigarillo graphic health warnings and quitting behaviors: a randomized clinical trial. *JAMA Netw Open.* 2025;8(8):e2526799. doi:10.1001/jamanetworkopen.2025.26799

eFigure. Effect of Condition on Quit Intentions (Panel A) and Quit Attempts (Panel B) at 21 Days, Adjusting for Baseline Values

eTable. Frequency of Reported Butting Out and Forgoing LCCs in the Last 7 Days

This supplementary material has been provided by the authors to give readers additional information about their work.

eFigure. Effect of Condition on Quit Intentions (Panel A) and Quit Attempts (Panel B) at 21 Days, Adjusting for Baseline Values. *P* values are based on a Wald chi-squared test.

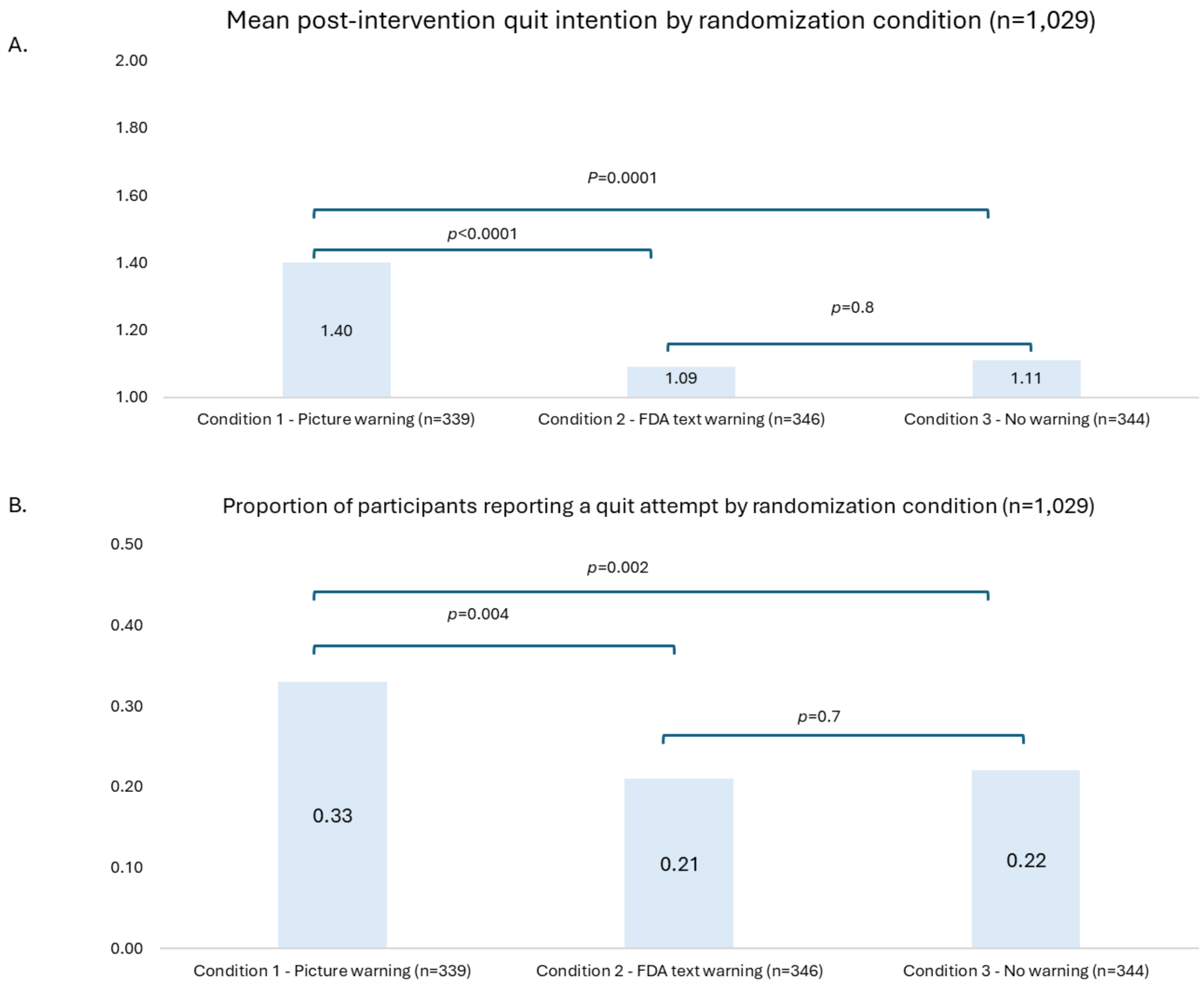

© 2025 Goldstein AO et al. *JAMA Network Open.*

eTable. Frequency of Reported Butting Out and Forgoing LCCs in the Last 7 Days

	**Day 7**	**Day 14**	**Day 21**
**Butting out little cigars**			
Condition 1: graphic health warning	2.08 (4.21) [n = 209]	1.72 (2.58) [n = 177]	2.04 (3.40) [n = 189]
Condition 2: text-only warning	2.11 (10.81) [n = 222]	2.05 (9.20) [n = 211]	2.06 (8.92) [n = 221]
Condition 3: no warning control	1.70 (3.96) [n = 209]	1.54 (4.35) [n = 203]	1.78 (3.74) [n = 211]
**Forgoing little cigars**			
Condition 1: graphic health warning	1.69 (2.81) [n = 307]	2.26 (16.24) [n = 286]	2.65 (7.36) [n = 311]
Condition 2: text-only warning	1.65 (9.01) [n = 305]	1.70 (7.72) [n = 300]	1.94 (7.95) [n = 317]
Condition 3: no warning control	1.28 (2.34) [n = 302]	1.24 (2.12) [n = 295]	1.40 (2.34) [n = 313]
**Butting out cigarillos**			
Condition 1: graphic health warning	3.22 (5.82) [n = 262]	2.54 (4.08) [n = 218]	2.45 (3.77) [n = 229]
Condition 2: text-only warning	2.31 (9.46) [n = 267]	2.44 (8.78) [n = 244]	2.49 (8.41) [n = 251]
Condition 3: no warning control	2.11 (3.67) [n = 255]	1.81 (3.00) [n = 241]	1.91 (3.36) [n = 254]
**Forgoing cigarillos**			
Condition 1: graphic health warning	2.16 (3.54) [n = 307]	3.44 (15.26) [n = 286]	3.04 (7.88) [n = 312]
Condition 2: text-only warning	1.99 (8.90) [n = 305]	1.77 (7.80) [n = 300]	2.36 (8.24) [n = 317]
Condition 3: no warning control	1.60 (3.14) [n = 302]	1.57 (3.02) [n = 295]	2.06 (6.28) [n = 313]

*Data reflects the mean (standard deviation) number of times an LCC was butted out or foregone.

© 2025 Goldstein AO et al. *JAMA Network Open.*
