# Supplementary material for: Little Cigar and Cigarillo Graphic Health Warnings and Quitting Behaviors: A Randomized Clinical Trial
Source: JAMA Netw Open. 2025 Aug 15;8(8):e2526799. doi: 10.1001/jamanetworkopen.2025.26799 (PMC12357197; doi:10.1001/jamanetworkopen.2025.26799)
Supplement: Supplement 3. — Data Sharing Statement [file jamanetwopen-e2526799-s003.pdf]

Data Sharing Statement

Goldstein. Little Cigar and Cigarillo Graphic Health Warnings and Quitting Behaviors. *JAMA Netw Open*. Published August 15, 2025. doi:10.1001/jamanetworkopen.2025.26799

Data
Additional Information: ClinicalTrials.gov identifier: NCT05849051
Data available: Yes
Data types: Deidentified participant data, Data dictionary
How to access data: For data requests, contact: aog@med.unc.edu
When available: With publication

Supporting Documents
Document types: Statistical/analytic code
How to access documents: https://cdn.clinicaltrials.gov/large-docs/51/NCT05849051/Prot_SAP_ICF_000.pdf?utm_source
When available: With publication

Additional Information
Who can access the data: As a supplemental file to main manuscript
Types of analyses: For any purpose
Mechanisms of data availability: As a supplemental file per above
Any additional restrictions: None
